# Risk of thrombocytopenic, haemorrhagic and thromboembolic disorders following COVID-19 vaccination and positive test: a self-controlled case series analysis in Wales

**DOI:** 10.1038/s41598-022-20118-6

**Published:** 2022-09-30

**Authors:** Fatemeh Torabi, Stuart Bedston, Emily Lowthian, Ashley Akbari, Rhiannon K. Owen, Declan T. Bradley, Utkarsh Agrawal, Peter Collins, Richard Fry, Lucy J. Griffiths, Jillian Beggs, Gareth Davies, Joe Hollinghurst, Jane Lyons, Hoda Abbasizanjani, Simon Cottrell, Malorie Perry, Richard Roberts, Amaya Azcoaga-Lorenzo, Adeniyi Francis Fagbamigbe, Ting Shi, Ruby S. M. Tsang, Chris Robertson, F. D. Richard Hobbs, Simon de Lusignan, Colin McCowan, Michael Gravenor, Colin R. Simpson, Aziz Sheikh, Ronan A. Lyons

**Affiliations:** 1 Population Data Science, Faculty of Medicine, Health & Life Science, Swansea University Medical School, Swansea University, Swansea, Wales, UK; 2 Centre for Public Health, School of Medicine Dentistry and Biomedical Sciences, Queen’s University Belfast, Belfast, UK; 3 Public Health Agency, Belfast, UK; 4 School of Medicine, University of St Andrews, St Andrews, UK; 5 Institute of Infection and Immunity, School of Medicine, Cardiff University, Cardiff, UK; 6 PPI, HDR UK BREATHE Hub, Edinburgh, UK; 7 Vaccine Preventable Disease Programme, Public Health Wales, Cardiff, UK; 8 Usher Institute, University of Edinburgh, Edinburgh, UK; 9 Nuffield Department of Primary Care Health Sciences, University of Oxford, Oxford, UK; 10 Department of Mathematics and Statistics, Strathclyde University, Glasgow and Public Health Scotland, Glasgow, UK; 11 School of Health, Victoria University of Wellington, Wellington, New Zealand; 12 HDR UK BREATHE Hub, Usher Institute, University of Edinburgh, Edinburgh, UK

**Keywords:** Public health, Experimental models of disease, Health care, Medical research

## Abstract

There is a need for better understanding of the risk of thrombocytopenic, haemorrhagic, thromboembolic disorders following first, second and booster vaccination doses and testing positive for SARS-CoV-2. Self-controlled cases series analysis of 2.1 million linked patient records in Wales between 7th December 2020 and 31st December 2021. Outcomes were the first diagnosis of thrombocytopenic, haemorrhagic and thromboembolic events in primary or secondary care datasets, exposure was defined as 0–28 days post-vaccination or a positive reverse transcription polymerase chain reaction test for SARS-CoV-2. 36,136 individuals experienced either a thrombocytopenic, haemorrhagic or thromboembolic event during the study period. Relative to baseline, our observations show greater risk of outcomes in the periods post-first dose of BNT162b2 for haemorrhagic (IRR 1.47, 95%CI: 1.04–2.08) and idiopathic thrombocytopenic purpura (IRR 2.80, 95%CI: 1.21–6.49) events; post-second dose of ChAdOx1 for arterial thrombosis (IRR 1.14, 95%CI: 1.01–1.29); post-booster greater risk of venous thromboembolic (VTE) (IRR-Moderna 3.62, 95%CI: 0.99–13.17) (IRR-BNT162b2 1.39, 95%CI: 1.04–1.87) and arterial thrombosis (IRR-Moderna 3.14, 95%CI: 1.14–8.64) (IRR-BNT162b2 1.34, 95%CI: 1.15–1.58). Similarly, post SARS-CoV-2 infection the risk was increased for haemorrhagic (IRR 1.49, 95%CI: 1.15–1.92), VTE (IRR 5.63, 95%CI: 4.91, 6.4), arterial thrombosis (IRR 2.46, 95%CI: 2.22–2.71). We found that there was a measurable risk of thrombocytopenic, haemorrhagic, thromboembolic events after COVID-19 vaccination and infection.

## Introduction

The COVID-19 vaccination programme began in Wales on 7th December 2020. By the end of 2021, there were three types of vaccine available in Wales: BNT162b2 mRNA (Pfizer-BioNTech, hereafter BNT162b2), ChAdOx1 nCoV-19 (Oxford-AstraZeneca, hereafter ChAdOx1) and mRNA-1273 (Moderna)^1^. The vaccine booster programme started in Wales on 25th October 2021, initially high risk vaccinated individuals were eligible to receive a booster at least 6 months after completion of their primary vaccine course^2^ this was updated later including all vaccinated individuals from three months after their initial dose of vaccine^3^. Boosters were all mRNA based with two types being offered in Wales: half a dose of mRNA-1273 (Moderna) or a complete dose of BNT162b2. Whilst the mRNA-1273 vaccine was made available in Wales from 7th April 2021, it was majorly administered in the booster programme, with only small numbers receiving it for their first and second dose. The vaccination delivery programme in Wales adhered to JCVI advice towards reducing the risk of serious outcomes while maximising protection in the population with continuous monitoring for hospitalisation rates and risk of death and serious outcomes^4,5^.

Indeed, all released vaccines have passed multiple safety and efficacy checks through each phase of clinical trials^6–9^. Clinical trials have limited ability to assess vaccine safety in relation to rare adverse outcomes^6–9^; hence, real-world evaluation of vaccine safety provides an opportunity to monitor incidence of rare adverse outcomes post vaccination. A media release in February 2021, about adverse reactions reported a possible association between ChAdOx1 and thrombotic events, specifically cerebral venous sinus thrombosis (CVST)^10^. In Wales, a set of rapid analyses were conducted in March 2021 to evaluate the number of rare clotting disorders following COVID-19 vaccination^11^. In June 2021, as part of a UK-wide study, we reported an increased risk of idiopathic thrombocytopenic purpura (ITP), arterial thromboembolism and haemorrhagic events associated with first dose of ChAdOx1 vaccine^12^. Similarly, a more recent study reporting results up to April 2021 in England reported an increased risk of thrombocytopenia, venous thromboembolism in the second week following first dose of ChAdOx1 vaccination and increased risk of ischaemic stroke in the third week following BNT162b2 vaccination^13^. We studied the risk of very rare cerebral venous thromboembolic (CVST) events as part of a UK-wide pooled analysis, showing a small increase of the risk of CVST post first dose of ChAdOx1. To date the risk of adverse clotting events been reported post first vaccination dose across UK nations^12–14^, the risk post second vaccinations and boosters are is under reported.

We used the Secure Anonymised Information Linkage (SAIL) Databank to analyse the risk of thrombocytopenic, haemorrhagic and thromboembolic events post first and second doses of ChAdOx1 and BNT162b2 and booster dose of BNT162b2 or mRNA-1273vaccines in the population of Wales. We evaluated vaccination safety and risk following each dose of COVID-19 vaccine, alongside the risks associated with SARS-CoV-2 infection.

### Added value of this work

This study is one of the first to examine the risk of all dosages of ChAdOx1 and BNT162b2 and booster on an array of serious bleeding and clotting events in the adult population of Wales, UK. We found that both COVID-19 vaccination and SARS-CoV-2 infection posed a measurable risk, while for VTE and haemorrhagic events there was a greater risk post infection compared to vaccination.

## Methods

We used a self-controlled case series (SCCS) approach to analyse, separately, the association of vaccination and reverse transcription‐polymerase chain reaction RT-PCR-confirmed SARS-CoV-2 infection with adverse haematological and venous events between 7th December 2020 and 31st December 2021. Anonymised individual-level, population-scale linked routinely collected electronic health record (EHR) data from multiple sources available in SAIL Databank were used^15,16^. The study cohort comprised of 3.4 million people residing in Wales. We constructed an electronic COVID-19 cohort with an anonymised linkage field in the SAIL Databank^17^. All those who were alive at the start of the study were checked against the eligibility criteria.

### Cohort eligibility

Our final sample included 2,062,144 individuals aged 16 or over who were registered with a primary care GP providing data to SAIL Databank in Wales (approximately 83% of all GPs in Wales providing data to SAIL Databank) who had valid Local Super Output Area (LSOA) code of residency in Wales as well as complete demographic and vaccination records (see Fig. 2 for consort diagram of cohort, Supplementary Table 1a for sample selection) and had sufficient follow up to 31 December 2021^17^ (Supplementary Table 2 for further details on each data source). LSOA code of residency was used to link individual records to the Welsh Index of Multiple Deprivation^18^ and determine deprivation status.

### Exposure

For analysing associations with a vaccine, we considered an individual exposed from the day they received their dose up to 28 days after vaccination. mRNA-1273was only included in our analysis when administered as a booster. First, second and third doses of mRNA-1273were excluded from our study due to insufficient numbers. Booster dose of vaccination was marked in the extract of Covid Vaccination Dataset (CVVD) and we used a sequential count to identify dosage including first, second and third dose of each vaccine.

For analysing associations with SARS-CoV-2 infection, we considered an individual exposed from the date the specimen was taken for the positive test, up to 28 days.

### Outcome events

The primary outcomes of interest were first clinical diagnosis of thrombocytopenic, haemorrhagic and thromboembolic events, including: venous thromboembolic (VTE), idiopathic thrombocytopenic purpura (ITP) and arterial thrombosis events in primary or secondary care electronic records. Our secondary outcomes were first hospitalisation for ischaemic stroke and myocardial infarction. To identify primary outcome events, we searched both primary and secondary care records (see Supplementary Table 3 for the list of International Classification of Diseases 10th revision (ICD-10) and Read (CTV2) codes used). Incident cases were defined as anyone having an event between 7th December 2020 (start of the vaccination programme in Wales) and 31st December 2021. To ensure the first incident was captured, individuals with an event in the year prior to the start of the vaccination period were removed from the analysis.

### Statistical analysis

**Figure 1:**
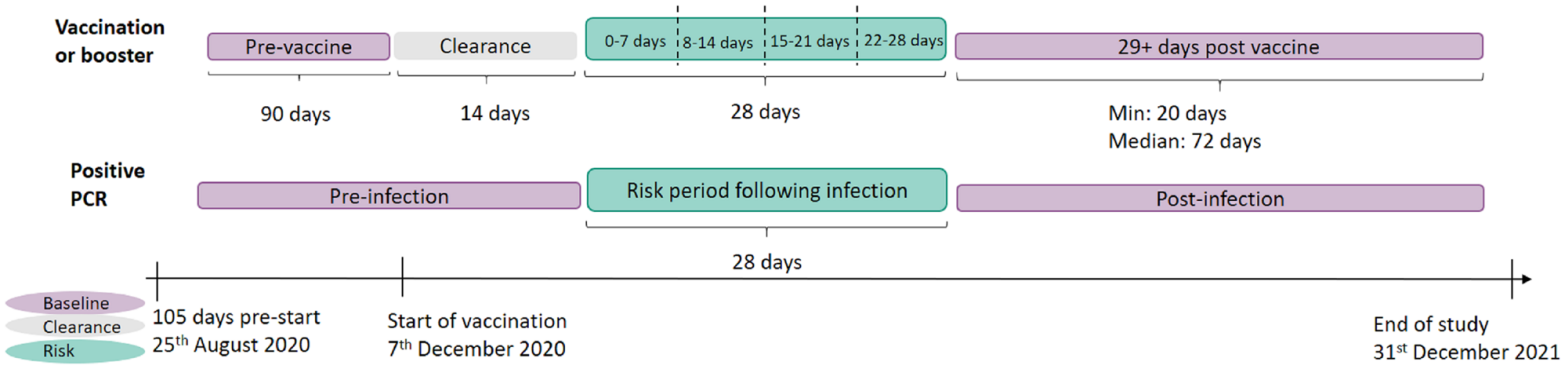
Self-controlled case series study design by vaccination status and positive PCR test status, separately.

We undertook a SCCS analysis of the people experiencing the incident outcome events during observation period^19–21^. We calculated the relative risk reported as incident rate ration (IRR) of these adverse events occurring in the 28 days after each dose of vaccination or SARS-CoV2 infection, compared against risk in baseline period (Fig. 1)^22^. As the inference for each outcome is based on inter-person changes, the SCCS method implicitly accounts for a range of covariates which remain unchanged during the observation window^21^. As the first vaccination in Wales was administered on 7th December 2020, considering a 90 days pre-risk interval and a 14-days clearance period, we started the study follow-up period 104 days prior to this date on 25th August 2020 or 104 days prior to an individual’s vaccination date, whichever was latest. We imposed a clearance period prior to each vaccination date as an earlier outcome event was considered highly likely to disrupt vaccination^23^. For the infection analysis, we did not impose a clearance period as an earlier outcome event was unlikely to disrupt future SARS-CoV2 infection. Vaccinated and tested cohorts were treated separately in this analysis.

For each dose of vaccination, the dates of administration were used as the start time of the 28 days exposure, respectively. The time between doses and booster was partitioned as a post-dose period and a 14 days clearance period. For those who had been only vaccinated with one dose, their time after the dose risk period was considered post-dose control. We ceased follow-up on an individual prior to the study end date if they died, were no longer registered with a primary care that provides data to SAIL (approximately 83% of all GPs in Wales providing data to SAIL Databank), or were no longer residing in Wales. The risk of developing an adverse event within the exposure windows are compared to the baseline risk of developing an event in pre-vaccination or ≥ 29 days post each vaccination dose (Fig. 1).

The model was fitted and IRRs were estimated using a conditional logistic regression, with the periods described in Fig. 1 being used as strata and their length as an offset^24^. Baseline intervals were used as the reference for vaccine and infection. We report estimates for baseline, clearance and risk intervals, along with 95% confidence intervals (CIs). R (version 5.3) was used to perform all analyses.

### Negative and positive controls

To check for potential confounding or bias, we considered events related to hip fractures as negative control outcomes^13^. Clinically, these events are unlikely to be directly associated with vaccination^25^. We also investigated the risk for combined anaphylaxis and adverse vaccine reactions (positive control) by extracting any hospital records for anaphylaxis and any recorded adverse reactions from the vaccination records of individuals. We used anaphylaxis as a positive control in this study, as those events are assumed to be highly associated with vaccination.

### Sensitivity analyses

We conducted two sets of sensitivity analyses: (I) excluding those who died within 90 days of an event, this was to handle any biases in our estimation based on the events which caused death^26^; and (II) excluding unvaccinated cases from analysis, this was to assess whether inclusion of unvaccinated cases affected our choice of risk period and estimated IRR^27^.

### Ethics approval and consent to participate

The data used in this study are accessed from the SAIL Databank (https://saildatabank.com/) at Swansea University, Swansea, UK. All proposals to use SAIL data are subject to review by an independent Information Governance Review Panel (IGRP). Before any data can be accessed, approval must be given by the IGRP. The IGRP gives careful consideration to each project to ensure proper and appropriate use of SAIL data which covers informed consent of participants where applicable. When access has been approved, it is gained through a privacy-protecting safe haven and remote access system referred to as the SAIL Gateway. SAIL has established an application process to be followed by anyone who would like to access data via SAIL https://www.saildatabank.com/application-process. This study has been approved by SAIL Information Governance Review Panel (IGRP) under application/project number 0911 and all research conducted in this study has been completed under the permission and approval of the IGRP.

## Results

**Figure 2:**
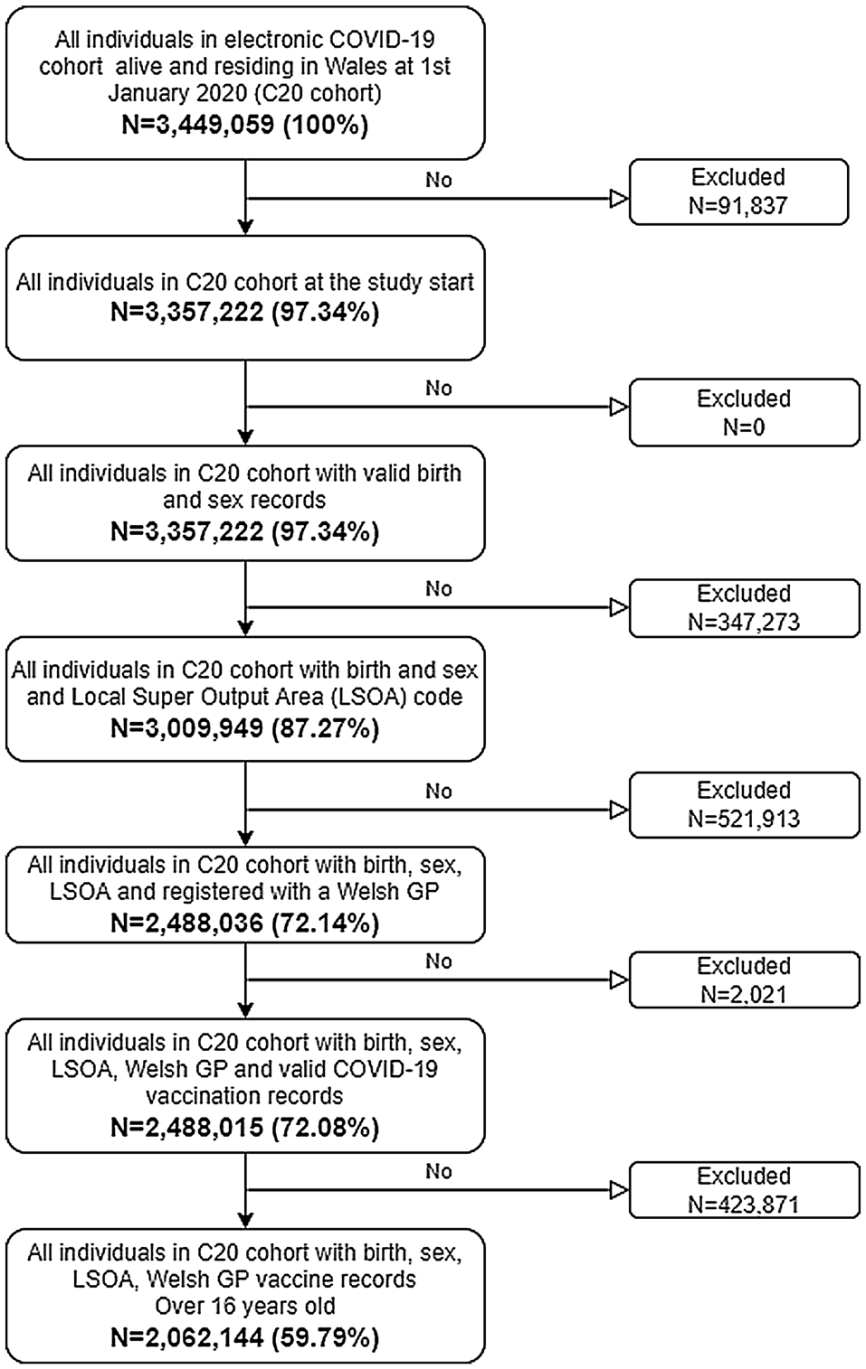
Consort diagram of cohort selection.

Our sample consisted of 2,062,144 individuals (mean age of 49 years, SD 19.56), contributing a total of 1,399,251 person-years of follow-up (See Fig. 2 and Supplementary Table 1 for sample selection summary). Of 2,062,144 individuals, 1,738,427 (84.3%) were vaccinated with two dosage primary schedule, 61,930 (3.0%) were vaccinated with only one dose, and 261,787 (12.7%) remained unvaccinated by 31st December 2021. We identified a total of 36,136 cases, of whom the majority 23,209 (64.2%) had received their booster dose (mRNA-1273 = 5410 and BNT162b2 = 17,799), with 1895 individuals receiving three vaccine doses (ChAdOx1 = 112, BNT162b2 = 1633) and 7111 individuals receivingtwo vaccine doses (ChAdOx1 = 5309, BNT162b2 = 1731) and 1,582 individuals receiving a single dose of vaccine (ChAdOx1 = 1254, BNT162b2 = 319) (Table 1 and Supplementary Table 4). In total, 6975 (19.3%) cases tested positive for SARS-CoV-2 during the observation window.

**Table 1:** Baseline characteristics of the study cohort (n = 36,136), stratified separately by vaccination status and PCR-confirmed SARS-CoV-2 infection status.

	COVID-19 vaccination status*									SARS-CoV2 infection status	
	Unvaccinated (n = 2339)	ChAdOx1 primary schedule			BNT162b2 primary schedule			BNT162b2 booster dose (n = 17,799)	mRNA-1273 booster dose** (n = 5410)	No positive PCR test (n = 29,161)	Positive PCR test (n = 6975)
		One dose only (n = 1254)	Two doses (n = 5309)	Three doses (n = 112)	One dose only (n = 319)	Two doses (n = 1731)	Three doses (n = 1633)				
**Incident category**											
Arterial Thrombosis	960 (41.0%)	573 (45.7%)	2562 (48.3%)	67 (59.8%)	89 (27.9%)	651 (37.6%)	707 (43.3%)	9229 (51.9%)	2364 (43.7%)	14,149 (48.5%)	3143 (45.1%)
Haemorrhagic events	252 (10.8%)	81 (6.5%)	431 (8.1%)	10 (8.9%)	47 (14.7%)	225 (13.0%)	184 (11.3%)	1890 (10.6%)	684 (12.6%)	3079 (10.6%)	748 (10.7%)
Idiopathic thrombocytopenic purpura	20 (0.9%)	10 (0.8%)	32 (0.6%)	< 5	< 5	34 (2.0%)	39 (2.4%)	86 (0.5%)	35 (0.6%)	207 (0.7%)	56 (0.8%)
Ischaemic Stroke	288 (12.3%)	189 (15.1%)	768 (14.5%)	Masked	39 (12.2%)	226 (13.1%)	127 (7.8%)	2085 (11.7%)	551 (10.2%)	3548 (12.2%)	762 (10.9%)
Myocardial Infarction	250 (10.7%)	145 (11.6%)	553 (10.4%)	Masked	Masked	165 (9.5%)	120 (7.3%)	1,650 (9.3%)	628 (11.6%)	2949 (10.1%)	624 (8.9%)
Thrombocytopenia	12 (0.5%)	7 (0.6%)	26 (0.5%)	< 5	< 5	20 (1.2%)	28 (1.7%)	65 (0.4%)	26 (0.5%)	158 (0.5%)	35 (0.5%)
Venous thromboembolic events	557 (23.8%)	249 (19.9%)	937 (17.6%)	14 (12.5%)	110 (34.5%)	410 (23.7%)	428 (26.2%)	2794 (15.7%)	1122 (20.7%)	5071 (17.4%)	1607 (23.0%)
**Sex**											
Female	1078 (46.0%)	603 (48.1%)	2701 (50.9%)	67 (59.8%)	155 (48.6%)	840 (48.5%)	748 (45.8%)	8564 (48.1%)	2271 (42.0%)	13,697 (47.0%)	3426 (49.1%)
Male	1261 (53.9%)	651 (51.9%)	2608 (49.1%)	45 (40.2%)	164 (51.4%)	891 (51.5%)	885 (54%)	9235 (51.9%)	3139 (58.0%)	15,464 (53.0%)	3549 (50.9%)
**Age band (years)**											
16–29	141 (6.0%)	10 (0.8%)	40 (0.8%)	< 5	41 (12.9%)	162 (9.4%)	21 (1.3%)	76 (0.4%)	89 (1.6%)	377 (1.3%)	211 (3.0%)
30–39	170 (7.3%)	36 (2.9%)	139 (2.6%)	< 5	41 (12.9%)	173 (10.0%)	30 (1.8%)	204 (1.1%)	177 (3.3%)	676 (2.3%)	313 (4.5%)
40–49	213 (9.1%)	87 (6.9%)	372 (7.0%)	< 5	41 (12.9%)	156 (9.0%)	76 (4.7%)	467 (2.6%)	507 (9.4%)	1397 (4.8%)	555 (8.0%)
50–59	360 (15.4%)	143 (11.4%)	756 (14.2%)	Masked	36 (11.3%)	190 (11.0%)	206 (12.6%)	1346 (7.6%)	1519 (28.1%)	3569 (12.2%)	1038 (14.9%)
60–69	386 (16.5%)	194 (15.5%)	837 (15.8%)	Masked	28 (8.8%)	188 (10.9%)	405 (24.8%)	3068 (17.2%)	1698 (31.4%)	5576 (19.1%)	1285 (18.4%)
70–79	475 (20.3%)	230 (18.3%)	967 (18.2%)	32 (28.6%)	87 (27.3%)	645 (37.3%)	578 (35.4%)	6609 (37.1%)	808 (14.9%)	8881 (30.5%)	1599 (22.9%)
80–89	424 (18.1%)	376 (30.0%)	1519 (28.6%)	40 (35.7%)	34 (10.7%)	162 (9.4%)	284 (17.4%)	4943 (27.8%)	453 (8.4%)	6806 (23.3%)	1463 (21.0%)
90+	170 (7.3%)	178 (14.2%)	679 (12.8%)	15 (13.4%)	11 (3.4%)	55 (3.2%)	33 (2.0%)	1086 (6.1%)	159 (2.9%)	1879 (6.4%)	511 (7.3%)
**Deprivation status**											
1 (most deprived)	690 (29.5%)	323 (25.8%)	1354 (25.5%)	11 (9.8%)	93 (29.2%)	464 (26.8%)	337 (20.6%)	3165 (17.8%)	1156 (21.4%)	5882 (20.2%)	1739 (24.9%)
2	563 (24.1%)	282 (22.5%)	1156 (21.8%)	28 (25.0%)	72 (22.6%)	343 (19.8%)	348 (21.3%)	3466 (19.5%)	1272 (23.5%)	6019 (20.6%)	1557 (22.3%)
3	382 (16.3%)	226 (18.0%)	1042 (19.6%)	28 (25.0%)	57 (17.9%)	323 (18.7%)	307 (18.8%)	3498 (19.7%)	1008 (18.6%)	5692 (19.5%)	1252 (17.9%)
4	379 (16.2%)	207 (16.5%)	884 (16.7%)	24 (21.4%)	48 (15.0%)	311 (18.0%)	290 (17.8%)	3744 (21.0%)	950 (17.6%)	5725 (19.6%)	1164 (16.7%)
5 (least deprived)	325 (13.9%)	216 (17.2%)	873 (16.4%)	21 (18.8%)	49 (15.4%)	290 (16.8%)	351 (21.5%)	3926 (22.1%)	1024 (18.9%)	5843 (20.0%)	1263 (18.1%)
**Number of previous COVID-19 tests**											
0	1712 (73.2%)	923 (73.6%)	3830 (72.1%)	79 (70.5%)	207 (64.9%)	1230 (71.1%)	1076 (65.9%)	14,259 (80.1%)	4215 (77.9%)	23,459 (80.4%)	4228 (60.6%)
1	311 (13.3%)	147 (11.7%)	749 (14.1%)	11 (9.8%)	55 (17.2%)	271 (15.7%)	273 (16.7%)	2127 (12.0%)	791 (14.6%)	3474 (11.9%)	1302 (18.7%)
2–4	230 (9.8%)	133 (10.6%)	513 (9.7%)	12 (10.7%)	39 (12.2%)	152 (8.8%)	222 (13.6%)	999 (5.6%)	331 (6.1%)	1716 (5.9%)	935 (13.4%)
5–9	69 (2.9%)	42 (3.3%)	163 (3.1%)	10 (8.9%)	8 (2.5%)	31 (1.8%)	46 (2.8%)	229 (1.3%)	52 (1.0%)	332 (1.1%)	328 (4.7%)
10+	17 (0.7%)	9 (0.7%)	54 (1.0%)	0 (0%)	10 (3.1%)	47 (2.7%)	16 (1.0%)	185 (1.0%)	21 (0.4%)	180 (0.6%)	182 (2.6%)

*Not shown are the 290 that were either on their first, second or third dose of mRNA-1273 under the primary vaccination schedule.

**Includes those who received half-dose of mRNA-1273.

The smallest proportion of cases (0.3%) were those receiving a third dose of ChAdOx1 vaccine as opposed to booster. Vaccination status was equally distributed among sex, in the majority of groups there was a notable 20% difference, between females (59.8%) receiving a third dose of ChAdOx1 compared to the males (40.2%). Those between 70 and 79 years and above had greater proportions of being vaccinated. The least deprived groups had a higher proportion of receiving a BNT162b2 booster (22.1%), while the proportion of unvaccinated were higher in the most deprived groups. The majority of cases did not have a recorded RT-PCR test for SARS-CoV-2 prior to experiencing an outcome event (Table 1).

### Thrombocytopenia (excluding ITP)

We observed no evidence for increased risk of thrombocytopenia events post first dose of ChAdOx1 or BNT162b2 vaccine. There was a suggestive increased risk, but imprecisely estimated due to low number of thrombocytopenia in 22–28 days post second dose of BNT162b2 (IRR 3.15, 95%CI 0.96, 10.32). Thrombocytopenia incidents were very rare post booster. We observed no evidence of change in risk of haemorrhagic events post booster dose of vaccines.

**Figure 3:**
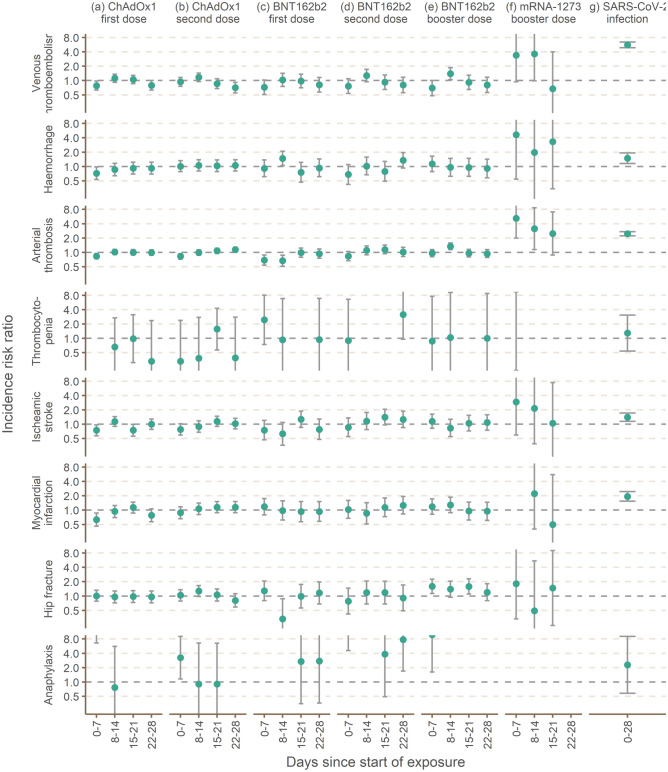
Incidence rate ratios (95% confidence intervals) for primary and secondary outcomes in 0–28 and 29 or more days after the first and second doses of ChAdOx1 (and BNF162b2 vaccines, and RT-PCR-confirmed SARS-CoV-2 infection, between 7 December 2020 and 31 December 2021.

There was a suggestive increased risk, but imprecisely estimated due to low number of thrombocytopenia events in 0–28 days post SARS-CoV-2 infection (IRR 1.29, 95%CI 0.54–3.07). See Fig. 3 and Table 2.

**Table 2:** Incidence rate ratios (95% confidence intervals) stratified by vaccination dose intervals, and separately by SARS-CoV-2 infection intervals (*P-value ≤ 0.05, **P-value ≤ 0.01).

		ChAdOx1 vaccine		BNT162b2 vaccine		PCR-confirmed SARS-CoV-2 infection		
Time period		Incidents	IRR (95% CI)	Incidents	IRR (95% CI)	Time period	Incidents	IRR (95% CI)
**Thrombocytopenia**								
	Baseline	105	1.00	39	1.00	Pre-infection	207	1.00
Dose 1	1–14 days-pre first dose clearance	< 10	0.92 (0.40, 2.1)	< 10	0.43 (0.06, 3.18)			
	0–7 days post first dose risk	0	–	< 10	2.44 (0.74, 8.05)			
	8–14 days post first dose risk	< 10	0.66 (0.16, 2.67)	< 10	0.93 (0.13, 6.84)			
	15–21 days post first dose risk	< 10	0.98 (0.31, 3.11)	< 10	0.94 (0.13, 6.90)			
	22–28 days post first dose risk	< 10	0.33 (0.05, 2.36)	–	–	0–28 days post-infection	< 10	1.29 (0.54, 3.07)
Dose 2	1–14 days-pre second dose clearance	< 10	0.54 (0.17, 1.69)	0	–			
	0–7 days post second dose risk	< 10	0.33 (0.05, 2.37)	< 10	0.90 (0.12, 6.56)			
	8–14 days post second dose risk	< 10	0.38 (0.05, 2.76)	0	–			
	15–21 days post second dose risk	< 10	1.56 (0.57, 4.26)	0	–			
	22–28 days post second dose risk	< 10	0.39 (0.05, 2.78)	< 10	3.15 (0.96, 10.32)*			
Dose 3	1–14 days-pre third dose clearance	0	–	< 10	2.45 (0.31, 19.28)			
	0–7 days post third dose risk	0	–	–	–	29+days post-infection	11	0.24 (0.11, 0.54)**
	8–14 days post third dose risk	0	–	–	–			
	15–21 days post third dose risk	0	–	–	–			
	22–28 days post third dose risk	0	–	–	–			
Booster		Moderna Half dose booster		BNT162b2 vaccine				
	1–14 days-pre booster clearance	< 10	Inf (0, Inf)	< 10	1.15 (0.24, 5.57)			
	0–7 days post booster risk	< 10	Inf (0, Inf)	< 10	0.87 (0.10, 7.60)			
	8–14 days post booster risk	0	–	< 10	1.04 (0.12, 9.12)			
	15–21 days post booster risk	0	–	< 10	1.00 (0.11, 8.77)			
	22–28 days post booster risk	0	–	0	–			
**Haemorrhage**								
	Baseline	1927	1.00	913	1.00	Pre-infection	3999	1.00
Dose 1	1–14 days-pre first dose clearance	78	0.71 (0.57, 0.89)**	33	0.68 (0.48, 0.96)*			
	0–7 days post first dose risk	43	0.72 (0.53, 0.98)**	24	0.91 (0.61, 1.37)			
	8–14 days post first dose risk	44	0.86 (0.64, 1.16)	33	1.47 (1.04, 2.08)*			
	15–21 days post first dose risk	47	0.92 (0.69, 1.23)	17	0.75 (0.47, 1.22)			
	22–28 days post first dose risk	47	0.92 (0.69, 1.23)	21	0.93 (0.61, 1.44)			
Dose 2	1–14 days-pre second dose clearance	64	0.61 (0.48, 0.79)*	29	0.63 (0.43, 0.91)**			
	0–7 days post second dose risk	57	1.01 (0.78, 1.32)	17	0.68 (0.42, 1.09)	0–28 days post-infection	100	1.49 (1.15, 1.92)**
	8–14 days post second dose risk	51	1.05 (0.80, 1.39)	22	1.02 (0.67, 1.57)			
	15–21 days post second dose risk	50	1.03 (0.78, 1.37)	17	0.79 (0.49, 1.28)			
	22–28 days post second dose risk	51	1.05 (0.80, 1.39)	29	1.35 (0.93, 1.96)			
Dose 3	1–14 days-pre third dose clearance	0	–	< 10	0.84 (0.33, 2.18)			
	0–7 days post third dose risk	0	–	< 10	–			
	8–14 days post third dose risk	0	–	< 10	1.10 (0.33, 3.60)			
	15–21 days post third dose risk	0	–	< 10	1.82 (0.70, 4.69)			
	22–28 days post third dose risk	0	–	< 10	0.70 (0.17, 2.91)	29+days post-infection	264	0.83 (0.68, 1.02)
Booster		Moderna Half dose booster		BNT162b2 vaccine				
	1–14 days-pre booster clearance	< 10	3.24 (0.39, 27.21)	62	1.40 (1.07, 1.84)**			
	0–7 days post booster risk	< 10	4.64 (0.54, 39.91)	30	1.13 (0.78, 1.65)			
	8–14 days post booster risk	< 10	1.98 (0.18, 21.91)	22	0.96 (0.62, 1.48)			
	15–21 days post booster risk	< 10	3.33 (0.34, 32.26)	22	0.95 (0.62, 1.47)			
	22–28 days post booster risk	0	–	21	0.91 (0.58, 1.42)			
**Venous thromboembolic (VTE)**								
	Baseline	3608	1.00	1533	1.00	Pre-infection	6635	1.00
Dose 1	1–14 days-pre first dose clearance	151	0.78 (0.66, 0.91)**	54	0.66 (0.50, 0.87)**			
	0–7 days post first dose risk	82	0.78 (0.63, 0.98)**	32	0.72 (0.51, 1.02)			
	8–14 days post first dose risk	100	1.12 (0.91, 1.36)	39	1.03(0.75, 1.41)			
	15–21 days post first dose risk	94	1.05 (0.85, 1.29)	37	0.97 (0.70, 1.35)			
	22–28 days post first dose risk	71	0.79 (0.62, 1.00)	31	0.81 (0.57, 1.16)			
Dose 2	1–14 days-pre second dose clearance	96	0.55 (0.46, 0.67)**	59	0.79 (0.61, 1.03)	0–28 days post-infection	525	5.63(4.91, 6.46)**
	0–7 days post second dose risk	89	0.94 (0.76, 1.16)	31	0.77 (0.54, 1.09)			
	8–14 days post second dose risk	95	1.17 (0.96, 1.44)	44	1.27 (0.94, 1.72)			
	15–21 days post second dose risk	70	0.86 (0.68, 1.09)	32	0.92 (0.65, 1.31)			
	22–28 days post second dose risk	58	0.71 (0.55, 0.93)	28	0.81 (0.56,1.17)			
Dose 3	1–14 days-pre third dose clearance	0	–	< 10	0.59 (0.28, 1.23)			
	0–7 days post third dose risk	0	–	< 10	–			
	8–14 days post third dose risk	0	–	< 10	1.12 (0.51, 2.46)			
	15–21 days post third dose risk	0	–	< 10	0.62 (0.22, 1.70)	29+ days post-infection	486	0.66 (0.57, 0.75)**
	22–28 days post third dose risk	0	–	< 10	0.46 (0.14, 1.48)			
Booster		Moderna Half dose booster		BNT162b2 vaccine				
	1–14 days-pre booster clearance	21	4.60 (1.35, 15.64)**	68	0.88 (0.68, 1.14)			
	0–7 days post booster risk	11	3.39 (0.94, 12.20)	31	0.70 (0.48, 1.01)*			
	8–14 days post booster risk	10	3.62 (0.99, 13.17)*	52	1.39 (1.04, 1.87)*			
	15–21 days post booster risk	< 10	0.67 (0.11, 3.99)	35	0.92 (0.65, 1.30)			
	22–28 days post booster risk	< 10	–	31	0.81 (0.56, 1.17)			
**Idiopathic thrombocytopenic purpura (ITP)**								
	Baseline	116	1.00	78	1.00	Pre-infection	261	1.00
Dose 1	1–14 days-pre first dose clearance	< 10	0.61 (0.22, 1.65)	< 10	0.25 (0.03, 1.78)			
	0–7 days post first dose risk	< 10	1.40 (0.57, 3.43)	< 10	2.80 (1.21, 6.49)*			
	8–14 days post first dose risk	< 10	1.31 (0.48, 3.56)	< 10	0.53 (0.07, 3.84)			
	15–21 days post first dose risk	< 10	0.98 (0.31, 3.08)	< 10	0.53 (0.07, 3.83)			
	22–28 days post first dose risk	< 10	0.97 (0.31, 3.07)	< 10	1.63 (0.51, 5.18)			
Dose 2	1–14 days-pre second dose clearance	< 10	0.51 (0.16, 1.62)	0	–			
	0–7 days post second dose risk	< 10	1.59 (0.64, 3.93)	< 10	0.47 (0.07, 3.40)			
	8–14 days post second dose risk	< 10	0.37 (0.05, 2.64)	0	–	0–28 days post-infection	13	2.46 (1.14, 5.33)*
	15–21 days post second dose risk	< 10	0.73 (0.18, 2.98)	0	–			
	22–28 days post second dose risk	< 10	1.76 (0.71, 4.36)	< 10	1.10 (0.27, 4.50)			
Dose 3	1–14 days-pre third dose clearance	0	–	< 10	0.33 (0.04, 2.84)			
	0–7 days post third dose risk	0	–	0	–			
	8–14 days post third dose risk	0	–	0	–			
	15–21 days post third dose risk	0	–	0	–	29+ days post-infection	12	0.29 (0.13, 0.61)**
	22–28 days post third dose risk	0	–	0	–			
Booster		Moderna Half dose booster		BNT162b2 vaccine				
	1–14 days-pre booster clearance	< 10	Inf (0, Inf)	< 10	0.77 (0.21, 2.75)			
	0–7 days post booster risk	0	–	< 10	0.89 (0.20, 4.01)			
	8–14 days post booster risk	0	–	< 10	0.50 (0.06, 3.84)			
	15–21 days post booster risk	< 10	Inf (0, Inf)	< 10	1.04 (0.23, 4.69)			
	22–28 days post third dose risk	0	–	0	–			
**Arterial Thrombosis**								
	Baseline	9501	1.00	3615	1.00	Pre-infection	17,648	1.00
Dose 1	1–14 days-pre first dose clearance	349	0.65 (0.58, 0.72)**	97	0.55 (0.45, 0.67)**			
	0–7 days post first dose risk	242	0.83 (0.73, 0.95)**	66	0.69 (0.54, 0.88)**			
	8–14 days post first dose risk	255	1.02 (0.90, 1.16)	55	0.67 (0.51, 0.87)**			
	15–21 days post first dose risk	249	1.00 (0.88, 1.14)	81	0.99 (0.79, 1.23)			
	22–28 days post first dose risk	247	0.99 (0.87, 1.13)	77	0.94 (0.75, 1.18)			
Dose 2	1–14 days-pre second dose clearance	335	0.67 (0.60, 0.75)	132	0.78 (0.65, 0.93)**			
	0–7 days post second dose risk	224	0.83 (0.72, 0.94)**	77	0.83 (0.67, 1.05)			
	8–14 days post second dose risk	229	0.99 (0.87, 1.13)	87	1.10 (0.89, 1.36)	0–28 days post-infection	718	2.46 (2.22, 2.71)**
	15–21 days post second dose risk	250	1.08 (0.95, 1.22)	91	1.15 (0.94, 1.42)			
	22–28 days post second dose risk	264	1.14 (1.01, 1.29)*	81	1.02 (0.82, 1.28)			
Dose 3	1–14 days-pre third dose clearance	< 10	0.35 (0.05, 2.52)	11	0.68 (0.37, 1.27)			
	0–7 days post third dose risk	0	–	12	0.69 (0.36, 1.27)			
	8–14 days post third dose risk	< 10	0.75 (0.10, 5.39)	10	1.31 (0.68, 2.51)			
	15–21 days post third dose risk	0	–	14	1.87 (1.07, 3.28)*			
	22–28 days post third dose risk	0	–	< 10	0.91 (0.42, 1.96)			
Booster		Moderna Half dose booster		BNT162b2 vaccine		29+ days post-infection	983	0.44 (0.40, 0.48)**
	1–14 days-pre booster clearance	48	6.77 (2.67, 17.17)**	229	0.84 (0.73, 0.97)*			
	0–7 days post booster risk	27	5.21 (2.00, 13.56)**	148	0.96 (0.81, 1.14)			
	8–14 days post booster risk	15	3.14 (1.14, 8.64)*	176	1.34 (1.15, 1.58)**			
	15–21 days post booster risk	12	2.49 (0.88, 7.07)	128	0.96 (0.80, 1.16)			
	22–28 days post third dose risk	< 10	–	125	0.94 (0.78, 1.14)			
**Ischaemic stroke**								
	Baseline	2410	1.00	876	1.00	Pre-infection	3799	1.00
Dose 1	1–14 days-pre first dose clearance	78	0.56 (0.45, 0.71)**	16	0.36 (0.22, 0.59)**			
	0–7 days post first dose risk	56	0.75 (0.57, 0.97)*	18	0.75 (0.47, 1.2)			
	8–14 days post first dose risk	73	1.14 (0.9, 1.44)	13	0.63 (0.36, 1.09)			
	15–21 days post first dose risk	48	0.75 (0.56, 0.99)*	26	1.27 (0.86, 1.88)			
	22–28 days post first dose risk	64	1 (0.78, 1.28)	16	0.78 (0.48, 1.28)			
Dose 2	1–14 days-pre second dose clearance	68	0.54 (0.43, 0.69)**	17	0.4 (0.25, 0.64)**			
	0–7 days post second dose risk	53	0.78 (0.59, 1.02)	20	0.86 (0.55, 1.35)			
	8–14 days post second dose risk	52	0.89 (0.68, 1.18)	23	1.16 (0.77, 1.76)	0–28 days post-infection	143	1.41 (1.15, 1.72)**
	15–21 days post second dose risk	67	1.15 (0.9, 1.47)	28	1.41 (0.97, 2.06)			
	22–28 days post second dose risk	60	1.03 (0.8, 1.33)	25	1.26 (0.85, 1.88)			
Dose 3	1–14 days-pre third dose clearance	< 10	3.52 (0.73, 16.95)	< 5	1.14 (0.36, 3.60)			
	0–7 days post third dose risk	< 10	2.94 (0.59, 14.68)	< 5	–			
	8–14 days post third dose risk	< 10	2.15 (0.39, 11.75)	< 5	1.09 (0.23, 5.04)			
	15–21 days post third dose risk	< 10	1.05 (0.15, 7.46)	< 5	0.95 (0.20, 4.44)			
	22–28 days post third dose risk	< 10	–	< 5	0.48 (0.06, 3.79)	29+ days post-infection	273	0.34 (0.29, 0.40)**
Booster		Moderna Half dose booster		BNT162b2 vaccine				
	1–14 days-pre booster clearance	0	–	44	0.75 (0.55, 1.04)			
	0–7 days post booster risk	0	–	38	1.15 (0.82, 1.62)			
	8–14 days post booster risk	0	–	24	0.83 (0.55, 1.27)			
	15–21 days post booster risk	0	–	30	1.05 (0.72, 1.54)			
	22–28 days post third dose risk	0	–	31	1.09 (0.75, 1.58)			
**Myocardial infarction**								
	Baseline	1950	1.00	760	1.00	Pre-infection	3799	1.00
Dose 1	1–14 days-pre first dose clearance	69	0.63 (0.49, 0.8)**	16	0.4 (0.25, 0.66)**			
	0–7 days post first dose risk	38	0.63 (0.46, 0.87)**	25	1.18 (0.79, 1.75)			
	8–14 days post first dose risk	48	0.94 (0.7, 1.25)	18	0.98 (0.62, 1.57)			
	15–21 days post first dose risk	58	1.14 (0.87, 1.47)	17	0.93 (0.57, 1.51)			
	22–28 days post first dose risk	40	0.78 (0.57, 1.06)	17	0.93 (0.58, 1.51)			
Dose 2	1–14 days-pre second dose clearance	57	0.57 (0.43, 0.74)**	13	0.35 (0.20, 0.60)**			
	0–7 days post second dose risk	48	0.88 (0.66, 1.17)	21	1.03 (0.67, 1.59)			
	8–14 days post second dose risk	50	1.07 (0.81, 1.41)	15	0.86 (0.51, 1.43)	0–28 days post-infection	123	1.94 (1.54, 2.46)**
	15–21 days post second dose risk	54	1.15 (0.88, 1.51)	20	1.14 (0.73, 1.79)			
	22–28 days post second dose risk	54	1.15 (0.88, 1.51)	22	1.26 (0.83, 1.93)			
Dose 3	1–14 days-pre third dose clearance	0	–	< 5	0.25 (0.03, 2.02)			
	0–7 days post third dose risk	0	–	< 5	Inf (0, Inf)			
	8–14 days post third dose risk	0	–	0	–			
	15–21 days post third dose risk	0	–	0	–	29+ days post-infection	180	0.38 (0.31, 0.47)**
	22–28 days post third dose risk	0	–	< 5	2.08 (0.62, 6.97)			
Booster		Moderna Half dose booster		BNT162b2 vaccine				
	1–14 days-pre booster clearance	< 10	2.85 (0.56, 14.57)	31	0.62 (0.43, 0.91)**			
	0–7 days post booster risk	–	–	33	1.18 (0.82, 1.71)			
	8–14 days post booster risk	< 10	2.21 (0.4, 12.14)	31	1.28 (0.88, 1.87)			
	15–21 days post booster risk	< 10	0.5 (0.05, 5.58)	23	0.96 (0.62, 1.47)			
	22–28 days post third dose risk	< 10	Inf (0, Inf)	23	0.95 (0.61, 1.46)			

### Haemorrhagic events

We observed a decreased risk of haemorrhagic events 0–7 days post first dose of ChAdOx1 (IRR 0.71 95% CI 0.57, 0.89). In the 8–14 days post first dose of BNT162b2, the risk was increased (IRR 1.47, 95% CI 1.04,2.08). There was no evidence of increased risk of haemorrhagic events post second dose of vaccines. We observed no evidence of change in risk of haemorrhagic events post booster dose of vaccines.

The risk of haemorrhagic events was elevated in 0–28 days post SARS-CoV-2 infection (IRR 1.49, 95% CI 1.15, 1.92). See Fig. 3 and Table 2.

### Venous thromboembolic events (VTE)

We observed a decreased risk of VTE events 8–14 days (IRR 0.78, 95%CI 0.63, 0.98) post first dose of ChAdOx1. We found no evidence of change in risk of VTE events post BNT162b2. There was no evidence of increased risk of VTE events post second dose of vaccines. There was a suggestive increased risk, but imprecisely estimated due to low number of events in 8–15 days post booster dose of mRNA-1273 (IRR 3.62, 95% CI 0.99, 13.17) as well as a booster dose of BNT162b2 (IRR 1.39, 95% CI 1.04, 1.87).

The risk of VTE events was elevated in 0–28 days post SARS-CoV-2 infection (IRR 5.63, 95% CI 4.91, 6.46). See Fig. 3 and Table 2.

### Idiopathic thrombocytopenic purpura (ITP)

We observed no evidence for increased risk of ITP events post first or second dose of ChAdOx1. The risk was elevated in 0–7 days post first dose of BNT162b2 vaccine (IRR 2.80, 95% CI 1.21, 6.49).

The risk of ITP events was elevated in 0–28 days post SARS-CoV-2 infection (IRR 2.46, 95% CI 1.14, 5.33) (Fig. 3 and Table 2).

### Arterial thrombosis

We observed a decreased risk of arterial thrombosis events 0–7 days post first dose of ChAdOx1 (IRR 0.65 95% CI 0.58, 0.72) and 0–7 days post second dose of ChAdOx1(IRR 0.83, 95%CI 0.72, 0.94). Similarly, the risk post first dose of BNT162b2 was decreased in 0–7 days (IRR 0.69, 95% CI 0.54, 0.88) and in 8–14 days (IRR 0.67, 95% CI 0.51, 0.87). The risk of arterial thrombosis events was elevated in 0–28 days post SARS-CoV-2 infection (IRR 2.46, 95% CI 2.22, 2.71) (Fig. 3 and Table 2).

### Secondary outcomes

#### Ischaemic stroke

We observed a decreased risk of ischaemic stroke events post first dose of ChAdOx1 in 0–7 days (IRR 0.75 95% CI 0.57, 0.97) and 15–21 days (IRR 0.75, 95%CI 0.56, 0.99). We found no evidence of change in the risk post first dose of BNT162b2 or booster. There was no evidence of increased risk of ischaemic stroke events post second dose of vaccines.

The risk of ischaemic stroke events was elevated in 0–28 days post SARS-CoV-2 infection (IRR 1.41, 95% CI 1.15, 1.72) (Fig. 3 and Table 2).

#### Myocardial infarction (MI)

We observed a decreased risk of MI events 0–7 days (IRR 0.63 95% CI 0.46, 0.87) post first dose of ChAdOx1. There were no evidence of change in the risk post second dose of ChAdOx1, first dose of BNT162b2 and booster.

The risk of MI events was elevated in 0–28 days post SARS-CoV-2 infection (IRR 1.94, 95% CI 1.54, 2.46) (Fig. 3 and Table 2).

### Negative and positive controls outcome

We found no evidence of change in risk of a hip fracture (negative control) in risk periods post first and second dose of ChAdOx1 and BNT162b2 vaccination or booster (supplementary Table 5). Risk of anaphylaxis was increased in risk periods post both vaccinations: mainly in 0–7 days post first dose of ChAdOx1 (IRR 11.87, 95% CI 6.56–21.47) post first dose of BNT162b2 (IRR 33.30, 95% CI 14.08–78.76) and in 0–7 days post second dose of ChAdOx1 (IRR 3.23, 95% CI 1.15–9.04) post first dose of BNT162b2 (IRR 14.62, 95% CI 4.56–46.85) (Supplementary Table 5).

### Sensitivity analyses

The first sensitivity analysis on (I) excluding those who died within 90 days of an event, showed a minimal change across all outcome groups with a slight increase of point estimates and confidence interval boundaries (Supplementary Table 6); (II) including only vaccinated cases, this gave practically identical results with slight change in confidence interval boundaries compared to our main analysis (Supplementary Table 7).

## Discussion

In this study of 2.1 million individuals in Wales, analysing the risk of thrombocytopenic, haemorrhagic, thromboembolic events post COVID-19 vaccination and infection. We identified 36,136 individuals who have experienced the outcomes during the study period.

For our primary outcome events, post-first dose of ChAdOx1, we observed suggestive evidence for increased risk of ITP and arterial thrombosis although this aligns with other studies^12,13^ because of limited number of observed events in our population this risk is imprecisely estimated and should be treated with caution. The risk was decreased for haemorrhagic, VTE and arterial thrombosis events. In the periods post-first dose of BNT162b2, our observations showed increased risk of haemorrhagic and ITP events. Post-second dose of ChAdOx1 there was an increased risk of arterial thrombosis but not post-second dose of BNT162bb2. Post-booster, for both mRNA-1273and BNT162b2, we observed an increased risk of VTE and arterial thrombosis. Post SARS-CoV-2 infection the risk was increased for haemorrhagic, VTE and arterial thrombosis events.

For our secondary outcomes, post-first dose of ChAdOx1, we observed a decreased risk of MI and ischeamic stroke events in the immediate 0–7 days post vaccination intervals. Post SARS-CoV-2 infection the risk for both MI and ischaemic stroke were elevated. To our knowledge, this is one of the first studies on evidence of the adverse bleeding and clotting risks following first and second dose of ChAdOx1 or BNT162b2 vaccination and boosters including mRNA-1273. Various studies, including ones from Scotland, England and Denmark have reported associations of first vaccination dose with adverse bleeding and clotting events^12–14,28,29^; however, none of the studies reported on risk post second dose and booster. Our results show that some risks persist post second and booster doses but there is considerable uncertainty due to the rarity of events. The analyses are strengthened by using a national linked data of the Welsh population which has considerable follow-up time of first, second and booster dose of vaccination. In addition, the study has high data completion and benefited from cross check on primary and secondary care dataset given the use of linkage across multiple routinely collected data sources. Our use of SCCS study design considering all vaccination dosage, showed robust findings for negative and positive controls for all risk groups.

Our study has several limitations including, limited power in accurately estimating the effect of thrombocytopenia and ITP due to the low number of events that are captured in this time period. We also use a sample that is smaller compared to other UK-wide studies. We analysed separately the risk post COVID-19 vaccination as primary exposure and post SARS-CoV-2 infection as secondary exposure; hence our ability in reporting the risk for the subset of cohort who might have experience both exposures in the same interval prior to their outcome is limited. Our ability in identification of cases and vaccination status is limited to what is recorded in routinely collected datasets, therefore, lags on recording the hospitalisation or transfer of records to general practice may have resulted in miss indentification of cases. Given the imprecision of some of our findings despite studying more than two million people we would like to see these analyses replicated in other countries and the data pooled in a meta-analysis. Although a number of risks were observed post-vaccination, the increase in risk post- SARS-CoV-2 infection appears to be of a greater magnitude and hence should not be a reason for delaying vaccination.

## Conclusions

Our study is one of the first to examine the risk of all dosages of ChAdOx1 and BNT162b2 and booster on an array of serious bleeding and clotting events. We found that both COVID-19 vaccination and SARS-CoV-2 infection posed a measurable risk, while for VTE and haemorrhagic events there was a greater risk post infection compared to vaccination. We did not find evidence of an increased risk of thrombocytopenia post vaccination or infection. Both BNT162n2 and mRNA-1273 boosters, shown consistent evidence for an increased risk of VTE in 8–14 days post booster. We did not find evidence for an increased risk of thrombocytopenia and ITP, however this may be due to these being rare events. ChAdOx1 had greater associations for ischaemic stroke, whereas BNT162b2 had greater associations for myocardial infarction. These findings have important considerations for health professionals, policy-makers, practitioners and the public in terms of understanding rare but serious bleeding and clotting events following COVID-19 vaccination.

## Supplementary Information

Supplementary Information.

## Data Availability

The Statistical Analysis plan of this work is available at: https://github.com/HDRUK/DaCVaP/blob/main/Wales/SAP/Workplan%20-%20Vaccine%20Safety.pdf. We also made the code and data to reproduce figures available at: https://github.com/HDRUK/DaCVaP/tree/main/Wales. The main patient-level data sources used in this study are available in the SAIL Databank at Swansea University, Swansea, UK, but as restrictions apply, they are not publicly available. All proposals to use SAIL data are subject to review by an independent Information Governance Review Panel (IGRP). Before any data can be accessed, approval must be given by the IGRP. The IGRP gives careful consideration to each project to ensure proper and appropriate use of SAIL data. When access has been granted, it is gained through a privacy protecting safe haven and remote access system referred to as the SAIL Gateway. SAIL has established an application process to be followed by anyone who would like to access data via SAIL at https://www.saildatabank.com/application-process/.

## References

[1] NHS-Wales. Get your COVID-19 vaccine|GOV.WALES [Internet]. [cited 2021 Aug 17]. Available from: https://gov.wales/get-your-covid-19-vaccine.

[2] JCVI. JCVI statement regarding a COVID-19 booster vaccine programme for winter 2021 to 2022—GOV.UK [Internet]. September 2021. [cited 2022 Feb 27]. p. 2021. Available from: https://www.gov.uk/government/publications/jcvi-statement-september-2021-covid-19-booster-vaccine-programme-for-winter-2021-to-2022/jcvi-statement-regarding-a-covid-19-booster-vaccine-programme-for-winter-2021-to-2022#fnref:6:1.

[3] COVID-19 vaccination booster|GOV.WALES [Internet]. [cited 2022 Feb 9]. Available from: https://gov.wales/covid-19-vaccination-booster.

[4] Optimising the COVID-19 vaccination programme for maximum short-term impact—GOV.UK [Internet]. [cited 2021 Aug 17]. Available from: https://www.gov.uk/government/publications/prioritising-the-first-covid-19-vaccine-dose-jcvi-statement/optimising-the-covid-19-vaccination-programme-for-maximum-short-term-impact.

[5] Government W. Vaccination saves lives Mae Brechu yn achub bywydau vaccination strategy for Wales. 2021 [cited 2022 Jan 11]; Available from: https://gov.wales/sites/default/files/publications/2021-01/vaccination-strategy-for-wales_3.pdf.

[6] Voysey M, Clemens SAC, Madhi SA, Weckx LY, Folegatti PM, Aley PK (2021). Safety and efficacy of the ChAdOx1 nCoV-19 vaccine (AZD1222) against SARS-CoV-2: An interim analysis of four randomised controlled trials in Brazil, South Africa, and the UK. Lancet.

[7] Polack FP, Thomas SJ, Kitchin N, Absalon J, Gurtman A, Lockhart S (2020). Safety and Efficacy of the BNT162b2 mRNA Covid-19 Vaccine. N. Engl. J. Med..

[8] Logunov DY, Dolzhikova IV, Shcheblyakov DV, Tukhvatulin AI, Zubkova OV, Dzharullaeva AS (2021). Safety and efficacy of an rAd26 and rAd5 vector-based heterologous prime-boost COVID-19 vaccine: An interim analysis of a randomised controlled phase 3 trial in Russia. Lancet.

[9] Baden LR, El Sahly HM, Essink B, Kotloff K, Frey S, Novak R (2021). Efficacy and safety of the mRNA-1273 SARS-CoV-2 vaccine. N. Engl. J. Med..

[10] Biotech J. Emergency use authorization (EUA) for an unapproved product review memorandum identifying information application type EUA (Event-driven EUA request) application number 27205 sponsor.

[11] Technical Advisory Cell. Technical advisory group high-level findings of clotting in. Tech Advis Cell. (March) (2021).

[12] Simpson CR, Shi T, Vasileiou E, Katikireddi SV, Kerr S, Moore E (2021). First-dose ChAdOx1 and BNT162b2 COVID-19 vaccines and thrombocytopenic, thromboembolic and hemorrhagic events in Scotland. Nat. Med..

[13] Hippisley-Cox J, Patone M, Mei XW, Saatci D, Dixon S, Khunti K (2021). Risk of thrombocytopenia and thromboembolism after covid-19 vaccination and SARS-CoV-2 positive testing: Self-controlled case series study. bmj.

[14] Patone M, Mei XW, Handunnetthi L, Dixon S, Zaccardi F, Shankar-Hari M (2021). Risks of myocarditis, pericarditis, and cardiac arrhythmias associated with COVID-19 vaccination or SARS-CoV-2 infection. Nat. Med..

[15] Lyons RA, Jones KH, John G, Brooks CJ, Verplancke J-P, Ford DV (2009). The SAIL databank: Linking multiple health and social care datasets. BMC Med. Inform. Decis. Mak..

[16] Ford DV, Jones KH, Verplancke JP, Lyons RA, John G, Brown G (2009). The SAIL databank: Building a national architecture for e-health research and evaluation. BMC Health Serv. Res..

[17] Lyons J, Akbari A, Torabi F, Davies GI, North L, Griffiths R (2020). Understanding and responding to COVID-19 in Wales: Protocol for a privacy-protecting data platform for enhanced epidemiology and evaluation of interventions. BMJ Open.

[18] StatsWales. Welsh index of multiple deprivation [Internet]. (2019) [cited 2022 Jul 13]. Available from: https://statswales.gov.wales/Catalogue/Community-Safety-and-Social-Inclusion/Welsh-Index-of-Multiple-Deprivation.

[19] Whitaker HJ, Farrington CP, Spiessens B, Musonda P (2006). Tutorial in biostatistics: The self-controlled case series method. Stat. Med..

[20] Self-controlled case series studies—Home [Internet]. [cited 2022 Feb 28]. Available from: https://sccs-studies.info/index.html.

[21] Farrington CP, Nash J, Miller E (1996). Case series analysis of adverse reactions to vaccines: A comparative evaluation. Am. J. Epidemiol..

[22] Petersen I, Douglas I, Whitaker H (2016). Self controlled case series methods: An alternative to standard epidemiological study designs. BMJ.

[23] Coronavirus (COVID-19) vaccines—NHS [Internet]. [cited 2021 Dec 3]. Available from: https://www.nhs.uk/conditions/coronavirus-covid-19/coronavirus-vaccination/coronavirus-vaccine/.

[24] R Core Team. R: A language and environment for statistical computing. R Foundation for Statistical Computing, Vienna, Austria [Internet]. cran. (2020) [cited 2021 Oct 21]. Available from: https://www.r-project.org/.

[25] Lipsitch M, Tchetgen Tchetgen E, Cohen T (2010). Negative controls: A tool for detecting confounding and bias in observational studies. Epidemiology.

[26] Paddy Farrington C, Anaya-Izquierdo K, Whitaker HJ, Hocine MN, Douglas I, Smeeth L (2012). Self-controlled case series analysis with event-dependent observation periods. J. Am. Stat. Assoc..

[27] Fonseca-Rodríguez O, Fors Connolly AM, Katsoularis I, Lindmark K, Farrington P (2021). Avoiding bias in self-controlled case series studies of coronavirus disease 2019. Stat. Med..

[28] Pottegård A, Lund LC, Karlstad Ø, Dahl J, Andersen M, Hallas J (2021). Arterial events, venous thromboembolism, thrombocytopenia, and bleeding after vaccination with Oxford-AstraZeneca ChAdOx1-S in Denmark and Norway: Population based cohort study. BMJ.

[29] Chui CSL, Fan M, Wan EYF, Leung MTY, Cheung E, Yan VKC (2022). Thromboembolic events and hemorrhagic stroke after mRNA (BNT162b2) and inactivated (CoronaVac) covid-19 vaccination: A self-controlled case series study. eClinicalMedicine.

