# Supplementary material for: Risk of thrombocytopenic, haemorrhagic and thromboembolic disorders following COVID-19 vaccination and positive test: a self-controlled case series analysis in Wales
Source: Sci Rep. 2022 Sep 30;12:16406. doi: 10.1038/s41598-022-20118-6 (PMC9524320; doi:10.1038/s41598-022-20118-6)
Supplement: Supplementary file 1 — Supplementary Information. [file 41598_2022_20118_MOESM1_ESM.pdf]

Supplementary
https://github.com/HDRUK/DaCVaP

List of abbreviations

Abbreviation term	Term
**GP**	general practitioner
**EHR**	electronic health record
**IRRs**	Incidence rate ratios
**ITP**	idiopathic thrombocytopenic purpura
**JCVI**	Joint Committee on Vaccination and Immunisation
**PCR**	polymerase chain reaction
**SAIL**	Secure Anonymised Information Linkage
**SCCS**	self-controlled case series
**SD**	Standard Deviation
**VE**	vaccine effectiveness
**VITT**	Vaccine Vaccine-Induced Immune Thrombotic Thrombocytopenia
**VTE**	venous thromboembolic
**WRRS**	Welsh Result Reporting Service

Supplementary Table 1a - Cohort eligibility
Please note: That the <5 counts that can be seen in the 'Events Diff' column are referring to records have been removed due to either incomplete or erroneous information. This means that these are not a privacy risk.

		Individuals			Events		
Step	**Sample criteria**	**n**	**Diff**	**(%)**	**n**	**Diff**	**(%)**
**0**	c20 total	3,449,059	NA	100	45,686	NA	100
**1**	in c20 at study start	3,357,222	-91837	97.34	45,605	-81	99.82
**2**	has wob and sex	3,357,222	0	97.34	45,605	0	99.82
**3**	has lsoa at study start	3,009,949	-347273	87.27	42,395	-3210	92.80
**4**	is registered with a gp	2,488,036	-521913	72.14	36,353	-6042	79.57
**5**	has no bad vacc records	2,486,015	-2021	72.08	36,315	-38	79.49
**6**	aged 16 years or older	2,062,144	-423871	59.79	36,136	-179	79.10

Supplementary Table 2 - Detail of all datasets used to derive research ready data assets for this study

Data sources

The following data sources were used to identify study population and outcome events:

- WLGP: Welsh Longitudinal General Practice – primary care GP data.
- PEDW: Patient Episode Dataset for Wales – secondary care hospital admissions.

We have also used linked patients data from other sources compiled in a research ready data assets:

- ADDD: Office for National Statistics (ONS) register of all deaths of Welsh residence. (Daily extract).
- ADDE: Office for National Statistics (ONS) register of all deaths of Welsh residence. (Extract).
- CDDS: COVID-19 Consolidated Deaths dataset.
- CVVD: Vaccination records from the Welsh Immunisation System (WIS). Covers date of vaccination, type of vaccination and also has a flag for adverse events immediately following vaccination (to be used by caution).
- PATD: Active COVID-19 PCR testing and results.
- WDDS: Welsh Dispensing Dataset.

Research Ready Data Assets (RRDAs):

- C19_COHORT20: follow-up to end of December 2021.
- C19_COHORT20_MORTALITY: A cleaned and organised table of mortality records, refreshed on a daily basis with coverage up to end of December 2021.
- C19_DERIVED_VACCINATION: A cleaned and organised table of vaccination records, refreshed on a daily basis with coverage up to end of December 2021.

Positive and Negative control definition and datasets:

Any hospitalisation for hip fracture is retrieved and used as a

For derivation of positive control we used PEDW dataset for any records of Anaphylaxis shock and combined it with any existing recorded adverse reactions to vaccine from CVSD dataset from all existing types of adverse reactions including: (1-local reaction, 2-syncope(faint), 3-Allergic reaction but not anaphylaxis 4-Anaphylactoid reaction and 5-Full anaphylaxis), only those recorded as anaphylactic reaction or full anaphylaxis were included and monitored for the cohort.

Supplementary Table 3a. ICD-10 codes for primary outcomes

ICD10	Description
**Thrombocytopenia**	
D695	Secondary thrombocytopenia
D694	Other primary thrombocytopenia
Idiopathic thrombocytopenic purpura (ITP)	
D693	Idiopathic thrombocytopenic purpura
**Venous thromboembolic events**	
I269	Pulmonary embolism without mention of acute cor pulmonale
I802	Phlebitis and thrombophlebitis of other deep vessels of lower extremities
H348	Other retinal vascular occlusions

I801	Phlebitis and thrombophlebitis of femoral vein
I81X	Portal vein thrombosis
I828	Embolism and thrombosis of other specified veins
I803	Phlebitis and thrombophlebitis of lower extremities, unspecified
I808	Phlebitis and thrombophlebitis of other sites
I800	Phlebitis and thrombophlebitis of superficial vessels of lower extremities
I260	Pulmonary embolism with mention of acute cor pulmonale
I809	Phlebitis and thrombophlebitis of unspecified site
I822	Embolism and thrombosis of vena cava
H342	Other retinal artery occlusions
I820	Budd-Chiari syndrome
H341	Central retinal artery occlusion
I829	Embolism and thrombosis of unspecified vein
I823	Embolism and thrombosis of renal vein
H349	Retinal vascular occlusion, unspecified
**Cerebral venous sinus thrombosis (CVST)**	
G08X	Intracranial and intraspinal phlebitis and thrombophlebitis
I676	Nonpyogenic thrombosis of intracranial venous system
I636	Cerebral infarction due to cerebral venous thrombosis, nonpyogenic
**Hemorrhage**	
R31X	Unspecified haematuria
K920	Haematemesis
T810	Haemorrhage and haematoma complicating a procedure, not elsewhere classified
R042	Haemoptysis
S065	Traumatic subdural haemorrhage
S066	Traumatic subarachnoid haemorrhage
I619	Intracerebral haemorrhage, unspecified
I620	Subdural haemorrhage (acute)(nontraumatic)
N029	Recurrent and persistent haematuriaunspecified
H431	Vitreous haemorrhage
I609	Subarachnoid haemorrhage, unspecified
N028	Recurrent and persistent haematuriaother
H113	Conjunctival haemorrhage
I629	Intracranial haemorrhage (nontraumatic), unspecified
I611	Intracerebral haemorrhage in hemisphere, cortical
I618	Other intracerebral haemorrhage
I615	Intracerebral haemorrhage, intraventricular
I610	Intracerebral haemorrhage in hemisphere, subcortical
J942	Haemothorax

R58X	Haemorrhage, not elsewhere classified
I614	Intracerebral haemorrhage in cerebellum
H356	Retinal haemorrhage
N421	Congestion and haemorrhage of prostate
I602	Subarachnoid haemorrhage from anterior communicating artery
I612	Intracerebral haemorrhage in hemisphere, unspecified
R048	Haemorrhage from other sites in respiratory passages
I601	Subarachnoid haemorrhage from middle cerebral artery
I613	Intracerebral haemorrhage in brain stem
I616	Intracerebral haemorrhage, multiple localized
I608	Other subarachnoid haemorrhage
I606	Subarachnoid haemorrhage from other intracranial arteries
I603	Subarachnoid haemorrhage from posterior communicating artery
I604	Subarachnoid haemorrhage from basilar artery
D683	Haemorrhagic disorder due to circulating anticoagulants
I607	Subarachnoid haemorrhage from intracranial artery, unspecified
D699	Haemorrhagic condition, unspecified
H313	Choroidal haemorrhage and rupture
I600	Subarachnoid haemorrhage from carotid siphon and bifurcation
I621	Nontraumatic extradural haemorrhage
R041	Haemorrhage from throat
D698	Other specified haemorrhagic conditions
**Arterial thromboembolic events**	
	currently no ICD10 codes identified for AT events
I60-I69	Note that these include 'chronic' occlusions from cholesterol etc and not just thromboembolic events but match to those in Nature paper
H34	
I20 to 124.9	IHD

Supplementary Table 3b. READ codes for primary outcomes

READ Code	Description
Thrombocytopenia	
42P2	Thrombocytopenia
D313.	Primary thrombocytopenia
D3133	[X]Essential thrombocytopenia NOS
D313y	Other specified primary thrombocytopenia
D313z	Primary thrombocytopenia NOS
D314	Secondary thrombocytopenia
D3141	Thrombocytopenia due to drugs
D314y	Other specified secondary thrombocytopenia
D314z	Secondary thrombocytopenia NOS
D315	Thrombocytopenia NOS
Dyu32	[X]Other primary thrombocytopenia
Idiopathic thrombocytopenic purpura (ITP)	
D3130	Idiopathic thrombocytopenic purpura
Venous thromboembolic events	
8CMWA	On deep vein thrombosis care pathway [s]
G801.	Deep vein phlebitis and thrombophlebitis of the leg [s]
G801B	Deep vein thrombophlebitis of the leg unspecified [s]
G801D	Deep vein thrombosis of lower limb [s]
G801F	Deep vein thrombosis of peroneal vein [s]
G801z	Deep vein phlebitis and thrombophlebitis of the leg NOS [s]
G8020	Thrombosis of vein of leg [s]
G80y4	Thrombophlebitis of the common iliac vein
G80y6	Thrombophlebitis of the external iliac vein
G401.	Pulmonary embolism [b]
G4011	Recurrent pulmonary embolism [b]
G81..	Portal vein thrombosis
G82..	Other venous embolism and thrombosis
G820.	Budd - Chiari syndrome (hepatic vein thrombosis)
G821.	Thrombophlebitis migrans
G822.	Embolism and thrombosis of the vena cava

G823.	Embolism and thrombosis of the renal vein
G82y.	Other embolism and thrombosis
G82z.	Embolism and thrombosis NOS
G82z0	Embolus of vein NOS
G82z1	Thrombosis of vein NOS
G82zz	Embolism and thrombosis NOS
F4238	Central retinal vein occlusion
8HTm.	Referral to deep vein thrombosis clinic

Cerebral venous sinus thrombosis (CVST)

G67A.	Cerebral vein thrombosis
F051z	Thrombosis of central nervous system venous sinus NOS
F053.	Thrombophlebitis of central nervous system venous sinuses
G676.	Nonpyogenic venous sinus thrombosis
F05..	Phlebitis and thrombophlebitis of intracranial sinuses
F050.	Embolism of central nervous system venous sinus
F0500	Embolism cavernous sinus
F0501	Embolism superior longitudinal sinus
F0502	Embolism lateral sinus
F0503	Embolism transverse sinus
F050z	Embolism central nervous system venous sinus NOS
F051.	Thrombosis of central nervous system venous sinuses
F0510	Thrombosis cavernous sinus
F0511	Thrombosis of superior longitudinal sinus
F0512	Thrombosis lateral sinus
F0513	Thrombosis transverse sinus
F052.	Phlebitis of central nervous system venous sinuses
F0520	Phlebitis cavernous sinus
F0521	Phlebitis of superior longitudinal sinus
F0522	Phlebitis lateral sinus
F0523	Phlebitis transverse sinus
F052z	Phlebitis of central nervous system venous sinus NOS
F0530	Thrombophlebitis of cavernous sinus
F0531	Thrombophlebitis of superior longitudinal venous sinus
F0532	Thrombophlebitis lateral venous sinus
F053z	Thrombophlebitis of central nervous system venous sinus NOS
F05z.	Phlebitis or thrombophlebitis of CNS venous sinus NOS

Haemorrhage

G61..	Intracerebral haemorrhage

G610.	Cortical haemorrhage
G611.	Internal capsule haemorrhage
G612.	Basal nucleus haemorrhage
G613.	Cerebellar haemorrhage
G614.	Pontine haemorrhage
G615.	Bulbar haemorrhage
G617.	Intracerebral haemorrhage, intraventricular
G619.	Lobar cerebral haemorrhage
G61X.	Intracerebral haemorrhage in hemisphere, unspecified
G61X0	Left sided intracerebral haemorrhage, unspecified
G61X1	Right sided intracerebral haemorrhage, unspecified
G61z.	Intracerebral haemorrhage NOS
G62..	Other and unspecified intracranial haemorrhage
G620.	Extradural haemorrhage – non-traumatic
G621.	Subdural haemorrhage – non-traumatic
G623.	Subdural haemorrhage NOS
G62z.	Intracranial haemorrhage NOS
2BB5.	O/E - retinal haemorrhages
2BB8.	O/E - vitreous haemorrhages
2DE7.	O/E - throat haemorrhage
C1542	Adrenal haemorrhage
F4045	Intraocular haemorrhage
F42y1	Superficial retinal haemorrhage
F42y3	Deep retinal haemorrhage
F42y4	Subretinal haemorrhage
F42y5	Retinal haemorrhage NOS
F436.	Choroidal haemorrhage and rupture
F4360	Unspecified choroidal haemorrhage
F4361	Expulsive choroidal haemorrhage
F436z	Choroidal haemorrhage or rupture NOS
F4C72	Conjunctival haemorrhage NOS
F4Ey0	Haemorrhage of eyelid
F4H41	Optic nerve sheath haemorrhage
F4K28	Vitreous haemorrhage
F4K7.	Retrobulbar haemorrhage
FyuH4	[X]Vitreous haemorrhage in diseases classified elsewhere
G8y0.	Haemorrhage NOS
Gyu61	[X]Other subarachnoid haemorrhage
Gyu62	[X]Other intracerebral haemorrhage
Gyu6F	[X]Intracerebral haemorrhage in hemisphere, unspecified
K1381	Renal artery haemorrhage

R048.	[D]Throat haemorrhage
R0631	[D]Pulmonary haemorrhage NOS
R09z0	[D]Umbilical bleeding
G60..	Subarachnoid haemorrhage
G601.	Subarachnoid haemorrhage from carotid siphon and bifurcation
G602.	Subarachnoid haemorrhage from middle cerebral artery
G603.	Subarachnoid haemorrhage from anterior communicating artery
G604.	Subarachnoid haemorrhage from posterior communicating artery
G605.	Subarachnoid haemorrhage from basilar artery
G60z.	Subarachnoid haemorrhage NOS
D3...	Clotting and bleeding disorders
D30..	Coagulation defects
D3z..	Clotting or bleeding disorder NOS

Arterial thromboembolic events

G63..	Precerebral arterial occlusion
G630.	Basilar artery occlusion
G631.	Carotid artery occlusion
G632.	Vertebral artery occlusion
G633.	Multiple and bilateral precerebral arterial occlusion
G634.	Carotid artery stenosis
G63y.	Other precerebral artery occlusion
G63y0	Cerebral infarct due to thrombosis of precerebral arteries
G63y1	Cerebral infarction due to embolism of precerebral arteries
G63z.	Precerebral artery occlusion NOS
G64..	Cerebral arterial occlusion
G640.	Cerebral thrombosis
G6400	Cerebral infarction due to thrombosis of cerebral arteries
G641.	Cerebral embolism
G6410	Cerebral infarction due to embolism of cerebral arteries
G64z.	Cerebral infarction NOS
G64z0	Brainstem infarction
G64z1	Wallenberg syndrome
G64z2	Left sided cerebral infarction
G64z3	Right sided cerebral infarction
G65..	Transient cerebral ischaemia
G650.	Basilar artery syndrome
G651.	Vertebral artery syndrome
G6510	Vertebro-basilar artery syndrome
G652.	Subclavian steal syndrome

G653.	Carotid artery syndrome hemispheric
G654.	Multiple and bilateral precerebral artery syndromes
G65y.	Other transient cerebral ischaemia
G65z.	Transient cerebral ischaemia NOS
G65z0	Impending cerebral ischaemia
G65z1	Intermittent cerebral ischaemia
G65zz	Transient cerebral ischaemia NOS
G66..	Stroke and cerebrovascular accident unspecified
G660.	Middle cerebral artery syndrome
G661.	Anterior cerebral artery syndrome
G662.	Posterior cerebral artery syndrome
G663.	Brain stem stroke syndrome
G664.	Cerebellar stroke syndrome
G665.	Pure motor lacunar syndrome
G666.	Pure sensory lacunar syndrome
G667.	Left sided CVA
G668.	Right sided CVA
G67..	Other cerebrovascular disease
G670.	Cerebral atherosclerosis
G671.	Generalised ischaemic cerebrovascular disease NOS
G6710	Acute cerebrovascular insufficiency NOS
G6711	Chronic cerebral ischaemia
G671z	Generalised ischaemic cerebrovascular disease NOS
G6730	Dissection of cerebral arteries, nonruptured
G677.	Occlusion/stenosis cerebral arts not result cerebral infarct
G6770	Occlusion and stenosis of middle cerebral artery
G6771	Occlusion and stenosis of anterior cerebral artery
G6772	Occlusion and stenosis of posterior cerebral artery
G6773	Occlusion and stenosis of cerebellar arteries
G6774	Occlusion+stenosis of multiple and bilat cerebral arteries
G67y.	Other cerebrovascular disease OS
G67z.	Other cerebrovascular disease NOS
G68..	Late effects of cerebrovascular disease
G680.	Sequelae of subarachnoid haemorrhage
G681.	Sequelae of intracerebral haemorrhage
G682.	Sequelae of other nontraumatic intracranial haemorrhage
G683.	Sequelae of cerebral infarction
G68W.	Sequelae/other + unspecified cerebrovascular diseases
G68X.	Sequelae of stroke,not specfd as h'morrhage or infarction
G6W..	Cereb infarct due unsp occlus/stenos precerebr arteries
G6X..	Cerebrl infarctn due/unspcf occlusn or sten/cerebrl artrs

G6y..	Other specified cerebrovascular disease
G74..	Arterial embolism and thrombosis
G740.	Embolism and thrombosis of the abdominal aorta
G741.	Embolism and thrombosis of the thoracic aorta
G742.	Embolism and thrombosis of an arm or leg artery
G7420	Embolism and thrombosis of the brachial artery
G7421	Embolism and thrombosis of the radial artery
G7422	Embolism and thrombosis of the ulnar artery
G7423	Embolism and thrombosis of an arm artery NOS
G7424	Embolism and thrombosis of the femoral artery
G7425	Embolism and thrombosis of the popliteal artery
G7426	Embolism and thrombosis of the anterior tibial artery
G7427	Embolism and thrombosis of the dorsalis pedis artery
G7428	Embolism and thrombosis of the posterior tibial artery
G7429	Embolism and thrombosis of a leg artery NOS
G742z	Peripheral arterial embolism and thrombosis NOS
G743.	Embolism and thrombosis of other and unspec parts aorta
G74y.	Embolism and thrombosis of other specified artery
G74y0	Embolism and/or thrombosis of the common iliac artery
G74y1	Embolism and/or thrombosis of the internal iliac artery
G74y2	Embolism and/or thrombosis of the external iliac artery
G74y3	Embolism and thrombosis of the iliac artery unspecified
G74y5	Embolism and thrombosis of the subclavian artery
G74y6	Embolism and thrombosis of the splenic artery
G74y7	Embolism and thrombosis of the axillary artery
G74y8	Embolism and thrombosis of the coeliac artery
G74y9	Embolism and thrombosis of the hepatic artery
G74yz	Embolism and thrombosis of other arteries NOS
G74z.	Arterial embolism and thrombosis NOS
F423.	Retinal vascular occlusion
F4230	Unspecified retinal vascular occlusion
F4231	Central retinal artery occlusion
F4232	Retinal arterial branch occlusion
F4233	Retinal microembolism
F4234	Hollenhorst plaque
F4235	Retinal partial arterial occlusion NOS
F4236	Amaurosis fugax
F4237	Retinal transient arterial occlusion NOS
G3...	Ischaemic heart disease
G30..	Acute myocardial infarction
G300.	Acute anterolateral infarction

G301.	Other specified anterior myocardial infarction
G3010	Acute anteroapical infarction
G3011	Acute anteroseptal infarction
G301z	Anterior myocardial infarction NOS
G302.	Acute inferolateral infarction
G303.	Acute inferoposterior infarction
G304.	Posterior myocardial infarction NOS
G305.	Lateral myocardial infarction NOS
G306.	True posterior myocardial infarction
G307.	Acute subendocardial infarction
G3070	Acute non-Q wave infarction
G3071	Acute non-ST segment elevation myocardial infarction
G308.	Inferior myocardial infarction NOS
G309.	Acute Q-wave infarct
G30B.	Acute posterolateral myocardial infarction
G30X.	Acute transmural myocardial infarction of unspecif site
G30X0	Acute ST segment elevation myocardial infarction
G30y.	Other acute myocardial infarction
G30y0	Acute atrial infarction
G30y1	Acute papillary muscle infarction
G30y2	Acute septal infarction
G30yz	Other acute myocardial infarction NOS
G30z.	Acute myocardial infarction NOS
G31..	Other acute and subacute ischaemic heart disease
G310.	Postmyocardial infarction syndrome
G310.	Dressler's syndrome
G3...	Arteriosclerotic heart disease
G311.	Preinfarction syndrome
G3110	Myocardial infarction aborted
G3111	Unstable angina
G3112	Angina at rest
G3113	Refractory angina
G3114	Worsening angina
G3115	Acute coronary syndrome
G311z	Preinfarction syndrome NOS
G312.	Coronary thrombosis not resulting in myocardial infarction
G31y.	Other acute and subacute ischaemic heart disease
G31y0	Acute coronary insufficiency
G31y1	Microinfarction of heart
G31y2	Subendocardial ischaemia
G31y3	Transient myocardial ischaemia

G31yz	Other acute and subacute ischaemic heart disease NOS
G35..	Subsequent myocardial infarction
G350.	Subsequent myocardial infarction of anterior wall
G351.	Subsequent myocardial infarction of inferior wall
G353.	Subsequent myocardial infarction of other sites
G35X.	Subsequent myocardial infarction of unspecified site
G36..	Certain current complication follow acute myocardial infarct
G360.	Haemopericardium/current comp folow acut myocard infarct
G361.	Atrial septal defect/curr comp folow acut myocardal infarct
G362.	Ventric septal defect/curr comp fol acut myocardal infarctn
G363.	Ruptur cardiac wall w'out haemopericard/cur comp fol ac MI
G364.	Ruptur chordae tendinae/curr comp fol acute myocard infarct
G365.	Rupture papillary muscle/curr comp fol acute myocard infarct
G38..	Postoperative myocardial infarction
G380.	Postoperative transmural myocardial infarction anterior wall
G381.	Postoperative transmural myocardial infarction inferior wall
G384.	Postoperative subendocardial myocardial infarction
G38z.	Postoperative myocardial infarction; unspecified
G3y..	Other specified ischaemic heart disease
G3z..	Ischaemic heart disease NOS
Gyu32	[X]Other forms of acute ischaemic heart disease
Gyu34	[X]Acute transmural myocardial infarction of unspecif site
Gyu36	[X]Subsequent myocardial infarction of unspecified site

Supplementary Table 4 - Number of cases stratified based on their vaccination status and dosage received by the end of 2021. Please note in Table 1 the two shaded groups are presented under Booster category.

No.	Total cases	VACC_COUNT	%	VACCINE_STATUS
**1**	36136	23140	64	D1 + D2 + Booster
**2**	36136	69	0.2	D1 + D2 + D3 + Booster
**3**	36136	1895	5.2	D1 + D2 + D3
**4**	36136	7111	19.7	D1 + D2
**5**	36136	1582	4.4	D1
**6**	36136	2339	6.5	UV

Supplementary Table 5- Negative and Positive control outcomes

Incidence rate ratios (95% confidence intervals) stratified by vaccination dose intervals, and separately by COVID-19 infection intervals.

		**ChAdOx1 vaccine**		**BNT162b2 vaccine**		**PCR-confirmed COVID-19 infection**
**Time period**	**Event Count**	**IRR (95% CI) – P-value**	**Event Count**	**IRR (95% CI) – P-value**	**Time period**	**IRR (95% CI) – P-value**
**Anaphylaxis**					Pre-infection	1.00
Baseline	51	1.00	18	1.00		
1-14 days-pre first dose clearance	<10	0.36(0.05,2.58)	0	---		
0-7 days post first dose risk	15	11.87(6.56,21.47)**	10	33.3 (14.08,78.76) **		
8-14 days post first dose risk	<10	0.77(0.11,5.58)	0	---		
15-21 days post first dose risk	0	--	<10	2.69 (0.35,20.42)		
22-28 days post first dose risk	0	--	<10	2.74 (0.36,20.89)		
1-14 days-pre second dose clearance	0	--	---	---		
0-7 days post second dose risk	<10	3.23(1.15,9.04)*	<10	14.62(4.56,46.85)**		
8-15 days post second dose risk	<10	0.9(0.12,6.55)	---	---	0-28 days post-infection	2.29 (0.58,9.04)
15-21 days post second dose risk	<10	0.9(0.12,6.53)	<10	3.85(0.49,30.01)		
22-28 days post second dose risk	0	---	<10	7.72(1.69,35.15) **		
1-14 days-pre third dose clearance	---	---	---	---		
0-7 days post third dose risk	---	---	---	---		
8-15 days post third dose risk	---	---	---	---		
15-21 days post third dose risk	---	---	---	---		
22-28 days post third dose risk	---	---	---	---	29+ days post-infection	1.14 (0.34,8.88)
**Booster**	Moderna or Moderna half vaccine		BNT162b2 vaccine			
1-14 days-pre second dose clearance	---	---	<10	0.48(0.03,7.57)		
0-7 days post second dose risk	<10	Inf (+inf,+inf) **	<10	9.80(1.62,59.35) **		
8-15 days post second dose risk	---	---	---	---		
15-21 days post second dose risk	---	---	---	---		
22-28 days post second dose risk	---	---	---	---		
**Hip fracture**						
Baseline	1916	1.00	548	1.00	Pre-infection	1.00
1-14 days-pre first dose clearance	36	0.34(0.24,0.47) **	<10	0.15(0.06,0.41)**		
0-7 days post first dose risk	58	1.01(0.78,1.32)	18	1.29(0.8,2.06)		
8-15 days post first dose risk	47	0.96(0.72,1.28)	<10	0.33(0.12,1.00)		
15-21 days post first dose risk	48	0.98(0.73,1.30)	12	0.99(0.56,1.76)		
22-28 days post first dose risk	47	0.96(0.72,1.28)	14	1.16(0.68,1.98)		
1-14 days-pre second dose clearance	59	0.60(0.46,0.78) **	<10	0.13(0.04,0.39) **		
0-7 days post second dose risk	55	1.04(0.79,1.36)	10	0.78(0.42,1.46)		

8-15 days post second dose risk	58	1.28(0.98,1.66)	13	1.19(0.68,2.06)	0-28 days post-infection	0.59 (0.47,0.74)**
15-21 days post second dose risk	48	1.06(0.79,1.41)	13	1.18(0.68,2.05)		
22-28 days post second dose risk	37	0.81(0.59,1.12)	10	0.91(0.49,1.7)		
1-14 days-pre third dose clearance	---	---	<10	0.8(0.21,3.03)		
0-7 days post third dose risk	---	---	<10	---		
8-15 days post third dose risk	---	---	<10	0.52(0.06,4.24)		
15-21 days post third dose risk	---	---	---	---		
22-28 days post third dose risk	---	---	---	---	29+ days post-infection	0.16 (0.14,0.19)
**Booster**	Moderna or Moderna half vaccine		BNT162b2 vaccine			
1-14 days-pre second dose clearance	13	0.27(0.15,0.47) **	41	1.60(1.13,2.26) *		
0-7 days post second dose risk	<10	1.82(0.33,10.15)	31	1.39(0.94,2.04)		
8-15 days post second dose risk	<10	0.49(0.04,5.47)	35	1.59(0.99,2.30) *		
15-21 days post second dose risk	<10	1.48(0.24,9.01)	0	---		
22-28 days post second dose risk	<10	---	27	1.2(0.80,1.81)		

* P-value ≤0.05
**P-value ≤0.01

Supplementary Table 6- Sensitivity analysis 1 – Excluding those who died within 90 days of the event

Incidence rate ratios (95% confidence intervals) stratified by vaccination dose intervals, and separately by COVID-19 infection intervals. This had minimal change on our results

		**ChAdOx1 vaccine**	**BNT162b2 vaccine**	**PCR-confirmed COVID-19 infection**	
**Time period**		**IRR (95% CI)**	**IRR (95% CI)**	**Time period**	**IRR (95% CI)**
**Thrombocytopenia**					
	Baseline	1.00	1.00	Pre-infection	1.00
**Dose 1**	1-14 days-pre first dose clearance	1.02(0.44,2.32)	0.46(0.06,3.36)		
	0-7 days post first dose risk	---	2.57(0.78,8.53)		
	8-14 days post first dose risk	0.73(0.18,2.94)	0.98(0.13,7.22)		
	15-21 days post first dose risk	1.09(0.34,3.43)	0.99(0.12,6.66)		
	22-28 days post first dose risk	0.36(0.05,2.61)	---	0-28 days post-infection	0.95 (0.33,2.79)
**Dose 2**	1-14 days-pre second dose clearance	0.58(0.18,1.83)	---		
	0-7 days post second dose risk	0.36(0.05,2.56)	1.04(0.14,7.62)		
	8-14 days post second dose risk	0.42(0.06,2.99)	--		
	15-21 days post second dose risk	1.69(0.62,4.62)	--		
	22-28 days post second dose risk	0.42(0.06,3.01)	3.15(0.96,10.32) *		
**Dose 3**	1-14 days-pre third dose clearance	---	2.36(0.30,18.57)		
	0-7 days post third dose risk	---	---	29+ days	0.38 (0.16,0.92) **

	8-14 days post third dose risk	---	---	post-infection	
	15-21 days post third dose risk	---	---		
	22-28 days post third dose risk	---	---		
**Booster**		Moderna Half dose booster	BNT162b2 vaccine		
	1-14 days-pre booster clearance	Inf (0,Inf)	1.13(0.23,5.41)		
	0-7 days post booster risk	Inf (0,Inf)	0.85(0.10,7.39)		
	8-14 days post booster risk	---	1.01(0.12,8.86)		
	15-21 days post booster risk	---	0.97(0.11,8.53)		
	22-28 days post booster risk	---	---		
**Haemorrhage**					
	Baseline	1.00	1.00	Pre-infection	1.00
**Dose 1**	1-14 days-pre first dose clearance	0.76(0.61,0.96) *	0.73(0.51,1.03)		
	0-7 days post first dose risk	0.72(0.53,0.99) *	0.98(0.65,1.47)		
	8-14 days post first dose risk	0.84(0.61,1.15)	1.57(1.11,2.23) **		
	15-21 days post first dose risk	0.95(0.70,1.27)	0.76(0.46,1.24)		
	22-28 days post first dose risk	0.95(0.70,1.27)	0.95(0.61,1.48)		
**Dose 2**	1-14 days-pre second dose clearance	0.62(0.48,0.80) **	0.66(0.45,0.96) *		
	0-7 days post second dose risk	1.01(0.77,1.32)	0.71(0.44,1.15)	0-28 days post-infection	1.54 (1.17,2.02) **
	8-14 days post second dose risk	1.00(0.74,1.34)	1.03(0.67,1.59)		
	15-21 days post second dose risk	0.98(0.73,1.31)	0.83(0.51,1.35)		
	22-28 days post second dose risk	0.98(0.73,1.31)	1.37(0.94,2.00) **		
**Dose 3**	1-14 days-pre third dose clearance	---	0.82(0.31,2.13)		
	0-7 days post third dose risk	---	---		
	8-14 days post third dose risk	---	0.71(0.17,2.98)		
	15-21 days post third dose risk	---	1.40(0.49,4.02)		
	22-28 days post third dose risk	---	0.67(0.16,2.83)	29+ days post-infection	0.94(0.75,1.17)
**Booster**		Moderna Half dose booster	BNT162b2 vaccine		
	1-14 days-pre booster clearance	3.24(0.39,27.21)	1.49(1.14,1.96) **		
	0-7 days post booster risk	4.64(0.54,39.91)	1.12(0.76,1.65)		
	8-14 days post booster risk	1.98(0.18,21.91)	0.97(0.62,1.51)		
	15-21 days post booster risk	3.33(0.34,32.26)	0.91(0.58,1.44)		
	22-28 days post booster risk	---	0.91(0.58,1.44)		
**Venous thromboembolic (VTE)**					
	Baseline	1.00	1.00	Pre-infection	1.00
**Dose 1**	1-14 days-pre first dose clearance	0.79(0.67,0.94) **	0.67 (0.50,0.89) **		
	0-7 days post first dose risk	0.77(0.61,0.97) *	0.71 (0.49,1.02)		
	8-14 days post first dose risk	1.05(0.84,1.30)	0.98 (0.69,1.38)		
	15-21 days post first dose risk	1.02(0.82,1.27)	1.01 (0.72,1.41)		
	22-28 days post first dose risk	0.75(0.58,0.97) *	0.89 (0.62,1.27)		
**Dose 2**	1-14 days-pre second dose clearance	0.56(0.45,0.69) **	0.84 (0.64,1.10)	0-28 days post-infection	5.32(4.57,6.19) **
	0-7 days post second dose risk	0.88(0.70,1.10)	0.74 (0.51,1.08)		
	8-14 days post second dose risk	1.05(0.84,1.31)	1.21 (0.88,1.67)		

	15-21 days post second dose risk	0.88(0.69,1.12)	0.90 (0.62,1.30)		
	22-28 days post second dose risk	0.71(0.54,0.93) **	0.77 (0.52,1.15)		
**Dose 3**	1-14 days-pre third dose clearance	---	0.56 (0.26,1.25)		
	0-7 days post third dose risk	---	---		
	8-14 days post third dose risk	---	1.06 (0.46,2.47)		
	15-21 days post third dose risk	---	0.33 (0.08,1.37)	29+ days post-infection	0.80(0.68,0.93) **
	22-28 days post third dose risk	---	0.51 (0.16,1.63)		
**Booster**		Moderna Half dose booster	BNT162b2 vaccine		
	1-14 days-pre booster clearance	4.8(1.41,16.37) **	0.90 (0.69,1.17)		
	0-7 days post booster risk	2.45(0.65,9.26)	0.61 (0.41,0.91) *		
	8-14 days post booster risk	3.63(1.00,13.22) *	1.35 (1.01,1.83) *		
	15-21 days post booster risk	0.67(0.11,3.99)	0.97 (0.68,1.37)		
	22-28 days post booster risk	---	0.77 (0.52,1.14)		
**Idiopathic thrombocytopenic purpura (ITP)**					
	Baseline	1.00	1.00	Pre-infection	1.00
**Dose 1**	1-14 days-pre first dose clearance	0.66(0.24,1.8)	0.26(0.04,1.91)		
	0-7 days post first dose risk	1.22(0.45,3.33)	2.50(1.00,6.24) *		
	8-14 days post first dose risk	1.43(0.53,3.90)	0.57(0.08,4.13)		
	15-21 days post first dose risk	1.07(0.34,3.38)	0.57(0.08,4.12)		
	22-28 days post first dose risk	1.07(0.34,3.36)	1.75(0.55,5.59)		
**Dose 2**	1-14 days-pre second dose clearance	0.55(0.17,1.76)	---		
	0-7 days post second dose risk	1.72(0.69,4.27)	0.50(0.07,3.64)		
	8-14 days post second dose risk	0.40(0.06,2.86)	---	0-28 days post-infection	1.54(0.64,3.70)
	15-21 days post second dose risk	0.79(0.19,3.23)	---		
	22-28 days post second dose risk	1.60(0.58,4.38)	1.18(0.29,4.82) - p-value=0.82		
**Dose 3**	1-14 days-pre third dose clearance	---	0.33(0.04,2.84) - p-value=0.31		
	0-7 days post third dose risk	---	---		
	8-14 days post third dose risk	---	---		
	15-21 days post third dose risk	---	---	29+ days post-infection	0.38(0.17,0.84) *
	22-28 days post third dose risk	---	---		
**Booster**		Moderna Half dose booster	BNT162b2 vaccine		
	1-14 days-pre booster clearance	Inf (0,Inf)	0.77(0.21,2.75)		
	0-7 days post booster risk	---	0.89(0.20,4.01)		
	8-14 days post booster risk	---	0.50(0.06,3.84)		
	15-21 days post booster risk	Inf (0,Inf)	1.04(0.23,4.69)		
	22-28 days post third dose risk	---	---		
**Arterial Thrombosis**					
	Baseline	1.00	1.00	Pre-infection	1.00
**Dose 1**	1-14 days-pre first dose clearance	0.69(0.62,0.77) **	0.58(0.48,0.71) **		
	0-7 days post first dose risk	0.81(0.70,0.93) **	0.72(0.56,0.92) **		
	8-14 days post first dose risk	0.99(0.87,1.13)	0.63(0.47,0.83) **		
	15-21 days post first dose risk	0.96(0.84,1.10)	0.96(0.76,1.21)		

	22-28 days post first dose risk	0.95(0.83,1.09)	0.89(0.70,1.13)		
**Dose 2**	1-14 days-pre second dose clearance	0.70(0.62,0.78) **	0.83(0.69,0.98) *		
	0-7 days post second dose risk	0.79(0.69,0.91) **	0.80(0.63,1.01)		
	8-14 days post second dose risk	0.92(0.80,1.06)	1.15(0.92,1.42)	0-28 days post-infection	1.98 (1.77,2.22) **
	15-21 days post second dose risk	1.02(0.89,1.17)	1.17(0.95,1.45)		
	22-28 days post second dose risk	1.13(0.99,1.28)	1.02(0.82,1.29)		
**Dose 3**	1-14 days-pre third dose clearance	0.35(0.05,2.51)	0.74(0.40,1.38)		
	0-7 days post third dose risk	---	---		
	8-14 days post third dose risk	0.75(0.10,5.37)	1.27(0.64,2.52)		
	15-21 days post third dose risk	---	1.73(0.95,3.15)		
	22-28 days post third dose risk	---	0.84(0.37,1.92)		
**Booster**		Moderna Half dose booster	BNT162b2 vaccine	29+ days post-infection	0.50 (0.45,0.56) **
	1-14 days-pre booster clearance	6.84(2.69,17.38) **	0.86(0.75,1.00) *		
	0-7 days post booster risk	4.53(1.73,11.90) **	0.96(0.80,1.14)		
	8-14 days post booster risk	3.14(1.14,8.66) *	1.25(1.05,1.48) **		
	15-21 days post booster risk	2.27(0.79,6.55)	0.90(0.74,1.10)		
	22-28 days post third dose risk	---	0.91(0.75,1.10)		
**Ischaemic stroke**					
	Baseline	1.00	1.00	Pre-infection	1.00
**Dose 1**	1-14 days-pre first dose clearance	0.62(0.49,0.78) **	0.40(0.25,0.66) **		
	0-7 days post first dose risk	0.67(0.50,0.91) **	0.75(0.46,1.23)		
	8-14 days post first dose risk	1.13(0.88,1.46)	0.66(0.37,1.16)		
	15-21 days post first dose risk	0.75(0.55,1.02)	1.21(0.79,1.85)		
	22-28 days post first dose risk	1.06(0.82,1.38)	0.55(0.29,1.02)		
**Dose 2**	1-14 days-pre second dose clearance	0.56(0.44,0.73) **	0.41(0.25,0.68) **		
	0-7 days post second dose risk	0.76(0.57,1.02)	0.86(0.54,1.37)		
	8-14 days post second dose risk	0.91(0.68,1.21)	1.17(0.76,1.81)	0-28 days post-infection	1.15 (0.9,1.46)
	15-21 days post second dose risk	1.10(0.85,1.43)	1.34(0.89,2.01)		
	22-28 days post second dose risk	0.95(0.71,1.26)	1.17(0.76,1.81)		
**Dose 3**	1-14 days-pre third dose clearance	---	1.32(0.43,4.12)		
	0-7 days post third dose risk	---	---		
	8-14 days post third dose risk	---	1.25(0.27,5.75)		
	15-21 days post third dose risk	---	1.09(0.24,5.06)		
	22-28 days post third dose risk	---	0.55(0.07,4.32)	29+ days post-infection	0.38 (0.32,0.47)**
**Booster**		Moderna Half dose booster	BNT162b2 vaccine		
	1-14 days-pre booster clearance	3.52(0.73,16.95)	0.76(0.54,1.06)		
	0-7 days post booster risk	2.94(0.59,14.68)	1.18(0.82,1.68)		
	8-14 days post booster risk	2.15(0.39,11.75)	0.77(0.49,1.21)		
	15-21 days post booster risk	1.05(0.15,7.46)	1.09(0.74,1.61)		
	22-28 days post third dose risk	---	1.00(0.67,1.50)		
**Myocardial infarction**					

	Time period	ChAdOx1 vaccine	BNT162b2 vaccine	PCR-confirmed COVID-19 infection Time period	IRR (95% CI)
	Baseline	1.00	1.00	Pre-infection	1.00
**Dose 1**	1-14 days-pre first dose clearance	0.68(0.53,0.87) - p-value=0	0.44(0.27,0.73) **		
	0-7 days post first dose risk	0.64(0.46,0.9) - p-value=0.01	1.19(0.78,1.80)		
	8-14 days post first dose risk	0.93(0.69,1.27) - p-value=0.65	0.84(0.49,1.42)		
	15-21 days post first dose risk	1.07(0.8,1.42) - p-value=0.65	0.78(0.45,1.35)		
	22-28 days post first dose risk	0.6(0.41,0.87) - p-value=0.01	0.78(0.45,1.35)		
**Dose 2**	1-14 days-pre second dose clearance	0.61(0.46,0.79) - p-value=0	0.37(0.22,0.65) **		
	0-7 days post second dose risk	0.8(0.58,1.09) - p-value=0.16	1.00(0.64,1.59)		
	8-14 days post second dose risk	1(0.74,1.35) - p-value=1	0.80(0.46,1.39)	0-28 days post-infection	1.61 (1.23,2.12) **
	15-21 days post second dose risk	1.07(0.8,1.44) - p-value=0.64	1.11(0.70,1.78)		
	22-28 days post second dose risk	1.17(0.88,1.54) - p-value=0.29	1.17(0.74,1.85)		
**Dose 3**	1-14 days-pre third dose clearance	---	0.25(0.03,2.03)		
	0-7 days post third dose risk	---	---		
	8-14 days post third dose risk	---	---		
	15-21 days post third dose risk	---	---	29+ days post-infection	0.46 (0.36,0.58) **
	22-28 days post third dose risk	---	2.09(0.62,7.02)		
**Booster**		Moderna Half dose booster	BNT162b2 vaccine		
	1-14 days-pre booster clearance	2.85(0.56,14.57)	0.64(0.43,0.94) *		
	0-7 days post booster risk	---	1.01(0.67,1.51)		
	8-14 days post booster risk	2.21(0.4,12.14)	1.03(0.67,1.58)		
	15-21 days post booster risk	0.5(0.05,5.58)	0.82(0.51,1.32)		
	22-28 days post third dose risk	Inf (0,Inf)	0.86(0.54,1.37)		

* P-value ≤0.05
**P-value ≤0.01

Supplementary Table 7- Sensitivity analysis 3 – Excluding unvaccinated cases

Incidence rate ratios (95% confidence intervals) stratified by vaccination dose intervals, and separately by COVID-19 infection intervals.

		ChAdOx1 vaccine	BNT162b2 vaccine	PCR-confirmed COVID-19 infection	
**Time period**		**IRR (95% CI)**	**IRR (95% CI)**	**Time period**	**IRR (95% CI)**
**Thrombocytopenia**					
	Baseline	1.00	1.00	Pre-infection	1.00
**Dose 1**	1-14 days-pre first dose clearance	0.92(0.4,2.1)	0.43(0.06,3.18)		
	0-7 days post first dose risk	---	2.44(0.74,8.05)		
	8-14 days post first dose risk	0.66(0.16,2.67)	0.93(0.13,6.84)		
	15-21 days post first dose risk	0.98(0.31,3.11)	---		

	22-28 days post first dose risk	0.33(0.05,2.36)	0.94(0.13,6.9)	0-28 days post-infection	3.08 (0.65,14.62)
**Dose 2**	1-14 days-pre second dose clearance	0.54(0.17,1.69)	---		
	0-7 days post second dose risk	0.33(0.05,2.37)	0.9(0.12,6.56)		
	8-14 days post second dose risk	0.38(0.05,2.76)	---		
	15-21 days post second dose risk	1.56(0.57,4.26)	---		
	22-28 days post second dose risk	0.39(0.05,2.78)	3.15(0.96,10.32)		
**Dose 3**	1-14 days-pre third dose clearance	---	2.45(0.31,19.28)		
	0-7 days post third dose risk	---	---	29+ days post-infection	---
	8-14 days post third dose risk	---	---		
	15-21 days post third dose risk	---	---		
	22-28 days post third dose risk	---	---		
**Booster**		Moderna Half dose booster	BNT162b2 vaccine		
	1-14 days-pre booster clearance	---	1.15(0.24,5.57)		
	0-7 days post booster risk	---	0.87(0.10,7.60)		
	8-14 days post booster risk	---	1.04(0.12,9.12)		
	15-21 days post booster risk	---	1.00(0.11,8.77)		
	22-28 days post booster risk	---	---		
**Haemorrhage**					
	Baseline	1.00	1.00	Pre-infection	1.00
**Dose 1**	1-14 days-pre first dose clearance	0.72(0.58,0.91) **	0.68(0.48,0.96) *		
	0-7 days post first dose risk	0.72(0.53,0.98) **	0.91(0.61,1.37)		
	8-14 days post first dose risk	0.86(0.64,1.16)	1.47(1.04,2.08) *		
	15-21 days post first dose risk	0.92(0.69,1.23)	0.75(0.47,1.22)		
	22-28 days post first dose risk	0.92(0.69,1.23)	0.93(0.61,1.44)		
**Dose 2**	1-14 days-pre second dose clearance	0.61(0.48,0.79) *	0.63(0.43,0.91) **		
	0-7 days post second dose risk	1.01(0.78,1.32)	0.68(0.42,1.09)	0-28 days post-infection	1.21 (0.56,2.61)
	8-14 days post second dose risk	1.05(0.80,1.39)	1.02(0.67,1.57)		
	15-21 days post second dose risk	1.03(0.78,1.37)	0.79(0.49,1.28)		
	22-28 days post second dose risk	1.05(0.80,1.39)	1.35(0.93,1.96)		
**Dose 3**	1-14 days-pre third dose clearance	---	0.84(0.33,2.18)		
	0-7 days post third dose risk	---	---		
	8-14 days post third dose risk	---	1.10(0.33,3.60)		
	15-21 days post third dose risk	---	1.82(0.70,4.69)		
	22-28 days post third dose risk	---	0.70(0.17,2.91)	29+ days post-infection	0.30(0.16,0.57)**
**Booster**		Moderna Half dose booster	BNT162b2 vaccine		
	1-14 days-pre booster clearance	3.24(0.39,27.21)	1.40(1.07,1.84) **		
	0-7 days post booster risk	4.64(0.54,39.91)	1.13(0.78,1.65)		
	8-14 days post booster risk	1.98(0.18,21.91)	0.96(0.62,1.48)		
	15-21 days post booster risk	3.33(0.34,32.26)	0.95(0.62,1.47)		
	22-28 days post booster risk	---	0.91(0.58,1.42)		
**Venous thromboembolic (VTE)**					
	Baseline	1.00	1.00	Pre-infection	1.00

**Dose 1**	1-14 days-pre first dose clearance	0.78 (0.66,0.91)**	0.66 (0.50,0.87) **		
	0-7 days post first dose risk	0.78 (0.63,0.98)**	0.72 (0.51,1.02)		
	8-14 days post first dose risk	1.12 (0.92,1.36)	1.03(0.75,1.41)		
	15-21 days post first dose risk	1.05 (0.86,1.29)	0.97 (0.70,1.35)		
	22-28 days post first dose risk	0.79 (0.62,1.00)	0.81 (0.57,1.16)		
**Dose 2**	1-14 days-pre second dose clearance	0.55 (0.46,0.67)**	0.79 (0.61,1.03)	0-28 days post-infection	7.99(5.79,11.01) **
	0-7 days post second dose risk	0.94 (0.76,1.16)	0.77(0.54,1.09)		
	8-14 days post second dose risk	1.17 (0.95,1.44)	1.27 (0.94,1.72)		
	15-21 days post second dose risk	0.86 (0.68,1.09)	0.92 (0.65,1.31)		
	22-28 days post second dose risk	0.71 (0.55,0.92)	0.81(0.56,1.17)		
**Dose 3**	1-14 days-pre third dose clearance	---	0.59 (0.28,1.23)		
	0-7 days post third dose risk	---	---		
	8-14 days post third dose risk	---	1.12 (0.51,2.46)		
	15-21 days post third dose risk	---	0.62 (0.22,1.70)	29+ days post-infection	0.23(0.16,0.34) **
	22-28 days post third dose risk	---	0.46 (0.14, 1.48)		
**Booster**		Moderna Half dose booster	BNT162b2 vaccine		
	1-14 days-pre booster clearance	4.60(1.35,15.64) **	0.88 (0.68,1.14)		
	0-7 days post booster risk	3.39(0.94,12.20)	0.70 (0.48,1.01) *		
	8-14 days post booster risk	3.62(0.99,13.17) *	1.39 (1.04,1.87) *		
	15-21 days post booster risk	0.67(0.11,3.99)	0.92 (0.65,1.30)		
	22-28 days post booster risk	---	0.81 (0.56,1.17)		
**Idiopathic thrombocytopenic purpura (ITP)**					
	Baseline	1.00	1.00	Pre-infection	1.00
**Dose 1**	1-14 days-pre first dose clearance	0.61(0.22,1.65)	0.25(0.03,1.78)		
	0-7 days post first dose risk	1.40(0.57,3.43)	2.80(1.21,6.49) *		
	8-14 days post first dose risk	1.31(0.48,3.56)	0.53(0.07,3.84)		
	15-21 days post first dose risk	0.98(0.31,3.08)	0.53(0.07,3.83)		
	22-28 days post first dose risk	0.97(0.31,3.07)	1.63(0.51,5.18)		
**Dose 2**	1-14 days-pre second dose clearance	0.51(0.16,1.62)	---		
	0-7 days post second dose risk	1.59(0.64,3.93)	0.47(0.07,3.40)		
	8-14 days post second dose risk	0.37(0.05,2.64)	---	0-28 days post-infection	Inf (-Inf,+Inf)
	15-21 days post second dose risk	0.73(0.18,2.98)	---		
	22-28 days post second dose risk	1.76 (0.71, 4.36)	1.10(0.27,4.50)		
**Dose 3**	1-14 days-pre third dose clearance	---	0.33(0.04,2.84)		
	0-7 days post third dose risk	---	---		
	8-14 days post third dose risk	---	---		
	15-21 days post third dose risk	---	---	29+ days post-infection	---
	22-28 days post third dose risk	0.61(0.22,1.65)	---		
**Booster**		Moderna Half dose booster	BNT162b2 vaccine		
	1-14 days-pre booster clearance	---	0.77(0.21,2.75)		
	0-7 days post booster risk	---	0.89(0.20,4.01)		
	8-14 days post booster risk	---	0.50(0.06,3.84)		
	15-21 days post booster risk	---	1.04(0.23,4.69)		

	22-28 days post third dose risk	---	---		
**Arterial Thrombosis**					
	Baseline	1.00	1.00	Pre-infection	1.00
**Dose 1**	1-14 days-pre first dose clearance	0.65(0.58,0.72) **	0.55(0.45,0.67) **		
	0-7 days post first dose risk	0.83(0.73,0.95) **	0.69(0.54,0.88) **		
	8-14 days post first dose risk	1.02(0.90,1.16)	0.67(0.51,0.87) **		
	15-21 days post first dose risk	1.00(0.88,1.14)	0.99(0.79,1.23)		
	22-28 days post first dose risk	0.99(0.87,1.13)	0.94(0.75,1.18)		
**Dose 2**	1-14 days-pre second dose clearance	0.67(0.60,0.75)	0.78(0.65,0.93) **		
	0-7 days post second dose risk	0.83(0.72,0.94) **	0.83(0.67,1.05)		
	8-14 days post second dose risk	0.99(0.87,1.13)	1.10(0.89,1.36)	0-28 days post-infection	5.99 (4.38,7.44) **
	15-21 days post second dose risk	1.08(0.95,1.22)	1.15(0.94,1.42)		
	22-28 days post second dose risk	1.14(1.01,1.29) *	1.02(0.82,1.28)		
**Dose 3**	1-14 days-pre third dose clearance	0.35(0.05,2.52)	0.68(0.37,1.27)		
	0-7 days post third dose risk	---	---		
	8-14 days post third dose risk	0.75(0.10,5.39)	1.31(0.68,2.51)		
	15-21 days post third dose risk	---	1.87(1.07,3.28) *		
	22-28 days post third dose risk	---	0.91(0.42,1.96)		
**Booster**		Moderna Half dose booster	BNT162b2 vaccine	29+ days post-infection	0.23 (0.18,0.29) **
	1-14 days-pre booster clearance	6.77(2.67,17.17) **	0.84(0.73,0.97) *		
	0-7 days post booster risk	5.21(2.00,13.56) **	0.96(0.81,1.14)		
	8-14 days post booster risk	3.14(1.14,8.64) *	1.34(1.15,1.58) **		
	15-21 days post booster risk	2.49(0.88,7.07)	0.96(0.80,1.16)		
	22-28 days post third dose risk	---	0.94(0.78,1.14)		
**Ischaemic stroke**					
	Baseline	1.00	1.00	Pre-infection	1.00
**Dose 1**	1-14 days-pre first dose clearance	0.56(0.45,0.71) **	0.36(0.22,0.59) **		
	0-7 days post first dose risk	0.75(0.57,0.97) *	0.75(0.47,1.2)		
	8-14 days post first dose risk	1.14(0.9,1.44)	0.63(0.36,1.09)		
	15-21 days post first dose risk	0.75(0.56,0.99) *	1.27(0.86,1.88)		
	22-28 days post first dose risk	1(0.78,1.28)	0.78(0.48,1.28)		
**Dose 2**	1-14 days-pre second dose clearance	0.54(0.43,0.69) **	0.4(0.25,0.64) **		
	0-7 days post second dose risk	0.78(0.59,1.02)	0.86(0.55,1.35)		
	8-14 days post second dose risk	0.89(0.68,1.18)	1.16(0.77,1.76)	0-28 days post-infection	2.74 (1.86,4.05)**
	15-21 days post second dose risk	1.15(0.9,1.47)	1.41(0.97,2.06)		
	22-28 days post second dose risk	1.03(0.8,1.33)	1.26(0.85,1.88)		
**Dose 3**	1-14 days-pre third dose clearance	3.52(0.73,16.95)	1.14(0.36,3.60)		
	0-7 days post third dose risk	2.94(0.59,14.68)	---		
	8-14 days post third dose risk	2.15(0.39,11.75)	1.09(0.23,5.04)		
	15-21 days post third dose risk	1.05(0.15,7.46)	0.95(0.20,4.44)		
	22-28 days post third dose risk	---	0.48(0.06,3.79)	29+ days post-infection	0.21 (0.14,0.31)**
**Booster**		Moderna Half dose booster	BNT162b2 vaccine		

	1-14 days-pre booster clearance	---	0.75(0.55,1.04)		
	0-7 days post booster risk	---	1.15(0.82,1.62)		
	8-14 days post booster risk	---	0.83(0.55,1.27)		
	15-21 days post booster risk	---	1.05(0.72,1.54)		
	22-28 days post third dose risk	---	1.09(0.75,1.58)		
**Myocardial infarction**					
	Baseline	1.00	1.00	Pre-infection	1.00
**Dose 1**	1-14 days-pre first dose clearance	0.63(0.49,0.8) **	0.4(0.25,0.66) **		
	0-7 days post first dose risk	0.63(0.46,0.87) **	1.18(0.79,1.75)		
	8-14 days post first dose risk	0.94(0.7,1.25)	0.98(0.62,1.57)		
	15-21 days post first dose risk	1.14(0.87,1.47)	0.93(0.57,1.51)		
	22-28 days post first dose risk	0.78(0.57,1.06)	0.93(0.58,1.51)		
**Dose 2**	1-14 days-pre second dose clearance	0.57(0.43,0.74) **	0.35(0.20,0.60) ***		
	0-7 days post second dose risk	0.88(0.66,1.17)	1.03(0.67,1.59)		
	8-14 days post second dose risk	1.07(0.81,1.41)	0.86(0.51,1.43)	0-28 days post-infection	3.82 (2.38,6.12) **
	15-21 days post second dose risk	1.15(0.88,1.51)	1.14(0.73,1.79)		
	22-28 days post second dose risk	1.15(0.88,1.51)	1.26(0.83,1.93)		
**Dose 3**	1-14 days-pre third dose clearance	---	0.25(0.03,2.02)		
	0-7 days post third dose risk	---	---		
	8-14 days post third dose risk	---	---		
	15-21 days post third dose risk	---	---	29+ days post-infection	0.18 (0.11,0.30) **
	22-28 days post third dose risk	---	2.08(0.62,6.97) - p-value=0.23		
**Booster**		Moderna Half dose booster	BNT162b2 vaccine		
	1-14 days-pre booster clearance	2.85(0.56,14.57)	0.62(0.43,0.91) **		
	0-7 days post booster risk	---	1.18(0.82,1.71)		
	8-14 days post booster risk	2.21(0.4,12.14)	1.28(0.88,1.87)		
	15-21 days post booster risk	0.5(0.05,5.58)	0.96(0.62,1.47)		
	22-28 days post third dose risk	Inf (0,Inf)	0.95(0.61,1.46)		

* P-value ≤0.05
**P-value ≤0.01
